# Supplementary material for: Cadmium binding by the F-box domain induces p97-mediated SCF complex disassembly to activate stress response programs
Source: Nat Commun. 2024 May 8;15:3894. doi: 10.1038/s41467-024-48184-6 (PMC11079001; doi:10.1038/s41467-024-48184-6)
Supplement: Supplementary file 1 — Supplementary Information [file 41467_2024_48184_MOESM1_ESM.pdf]

Supplementary Information

Figure S1

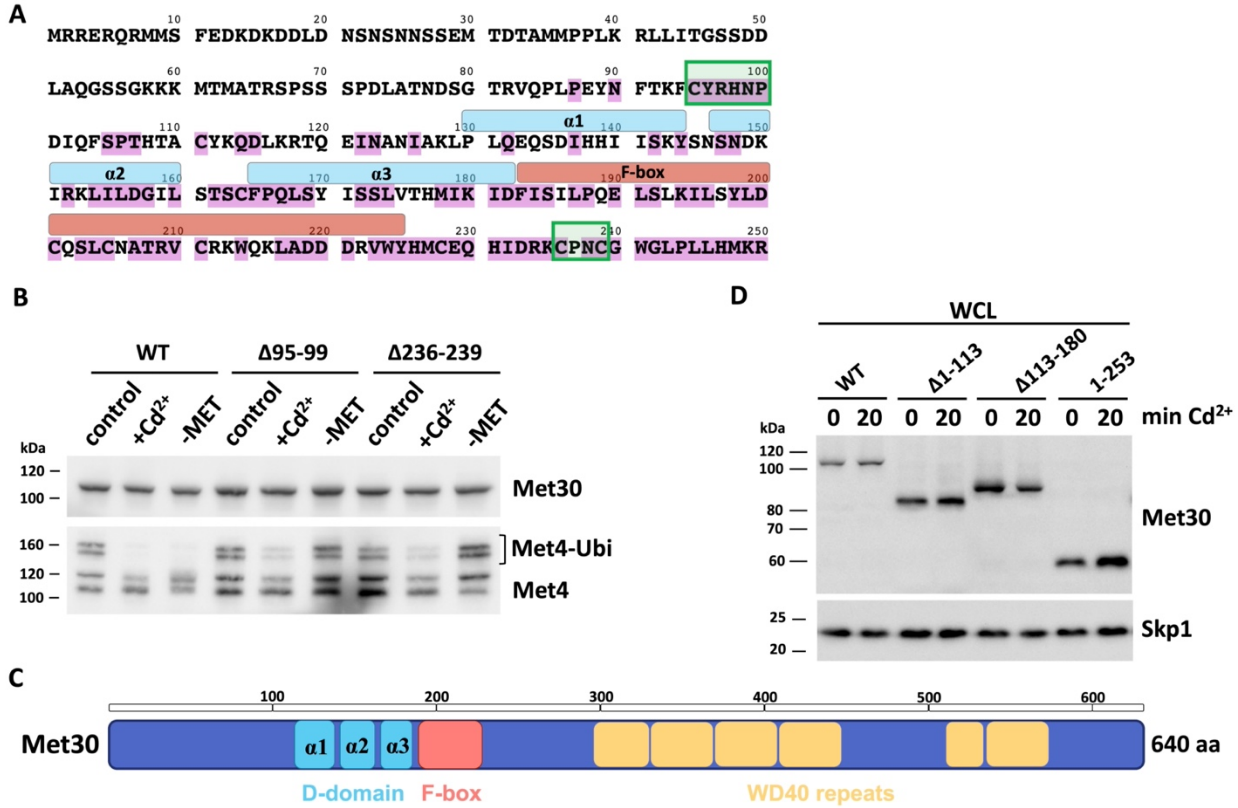

A) Conservation ranking of amino acid residues 1-250 in Met30. Highly conserved amino acids are highlighted in magenta. Dimerization domain (D-Domain) aa 129-181 in light blue, F-box motif aa 183-226 in red. Green boxes indicate proposed metal binding motifs (aa 95-99 and aa 236-239). "The ConSurf Server" (https://consurf.tau.ac.il) was consulted to determine conservation raking of amino acids. B) Proposed metal binding motifs seem to be involved in the methionine sensing pathway but not cadmium stress. Strains expressing endogenous ^{12xMyc}Met30 WT, Δ95-99, or Δ236-239 were cultured at 30°C in YEPD medium and shifted to defined media without methionine or treated with 100 µM $CdCl_2$ for 30 min respectively. Whole cells lysates (WCL) of samples were analyzed by Western blot using a Met4 antibody to follow the ubiquitylation status of Met4 and a MYC antibody was used to detect Met30. C) Schematic of the F-box protein Met30. Dimerization domain (D- Domain) aa 129-181 in light blue, F-box motif aa 183-226 in red, WD40 repeats aa 297-586: I 297- 336, II 337-376, III 377-416, IV 416-457, V 524-546, VI 546-586 in yellow. "prosite.expasy.org" was used to define motifs. D) Characterization of a potential cadmium sensing domain in Met30. D) Expression of Met30 truncation and internal deletion mutants. Protein levels of Met30 and Skp1 in cells expressing reintroduced ^{12xMyc}Met30 WT and mutants from a single copy vector respectively under normal and cadmium exposed conditions. Cells were cultured at 30̊C in SD - LEU medium and treated with 100 µM $CdCl_2$, and samples were harvested after 20 minutes of exposure. Native whole cell lysates of samples were analyzed by Western blot using a Skp1 antibody and a MYC antibody was used to detect Met30.

Figure S2

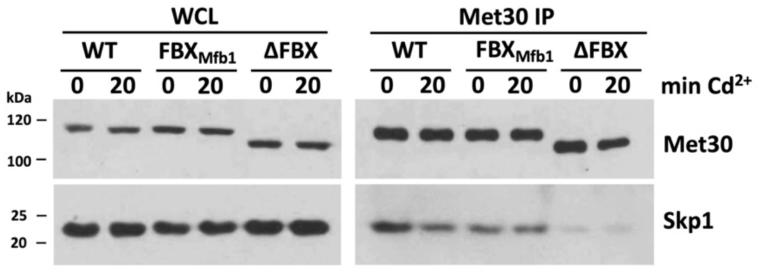

Cells expressing reintroduced 12xMycMet30 WT, entire F-box deletion, or with the F-box motif of Mfb1 were cultured at 30˚C in SC -LEU medium and treated with 100 µM $CdCl_2$, and samples were harvested after 20 minutes of exposure. Native whole cell lysates were prepared and 12xMycMet30 was immunoprecipitated (Met30 IP) and co-precipitated Skp1 was analyzed by Western blot.

Figure S3

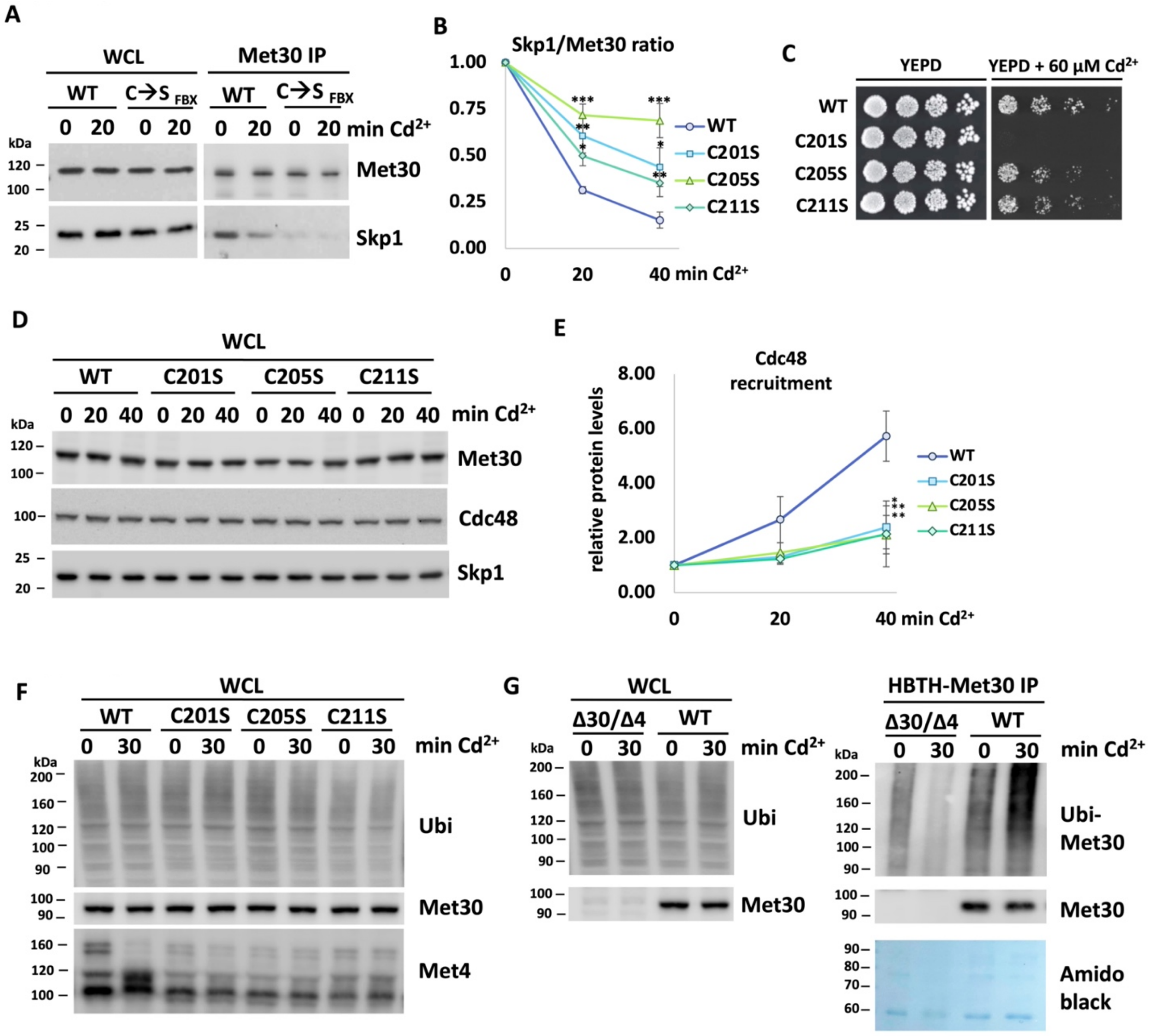

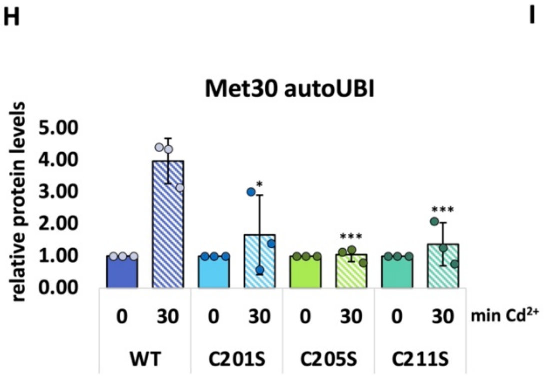

I

Met30 intra X-links	Control	Cadmium
K58-K60	37	5
K128-K150	5	1
K216-K283	2	
K275-K283	2	1
K283-K290	9	2
K290-K354	4	
K346-K354	3	5
K346-K362	5	2
K346-K468	2	
K354-K393	7	2
K354-K405	1	2
K354;K362-K393	6	
K362-K405	3	4
K362-K457	2	
K393-K405		2
K457-K540;K543	11	2
Total X-links	99	28

Total Met30 peptide count	488	580

A) Cells expressing endogenous ^{12Myc}Met30 WT F-box motif or mutation of C201S, C205S & C211S (CS) in the F-box motif were cultured at 30°C in YEPD medium and treated with 100 µM CdCl2 for 20 minutes. Native whole cells lysates (WCL) of samples were prepared and ^{12xMyc}Met30 was immunoprecipitated (Met30 IP) and co-precipitated Skp1 was analyzed by Western blot. B) Densitometric analysis of Western blot band intensities of immunoprecipitations in figure 3B. For quantifications the signal intensity for WT Met30 variant at time point 0 was set to 1 and Skp1 signals were normalized to ^{12xMyc}Met30 to quantify Met30 dissociation from the core ligase (n=3 independent experiments), data are represented as mean ±SD, *p* values calculated by two-tailed student's t-test: p*< 0.1, p**<0.05, p***< 0.01. C) Strains shown in figure 3 were cultured to logarithmic growth phase, cells were counted, and serial dilutions spotted onto YEPD plates supplemented with or without 60 µM $CdCl_2$. Plates were incubated for two days at 30°C (n=3). D) Whole cells lysates of Met30 Immunoprecipitation shown in figure 4A. E) Densitometric analysis of Western blot band intensities shown in figure 4A. For quantifications the signal intensities for each Met30 variant at time point 0 were set to 1 and Cdc48 signals were normalized to ^{12xMyc}Met30 to quantify segregase recruitment to SCF^{Met30} (n=3 independent experiments), data are represented as mean ±SD, *p* values calculated by two-tailed student's t-test: p*< 0.1, p**<0.05, p***< 0.01. F) Cadmium-induced autoubiquitylation of Met30 is decreased in single cysteine mutants. Denaturing whole cells lysates of Ni^{2+}-NTA pulldowns shown in figure 4B. G) Cells in which *met30* and *met4* are deleted, were used as a control to define unspecific ubiquitin background precipitation. Those and cells expressing ^{HBTH}Met30 were cultured at 30°C in YEPD medium and treated with 100 µM $CdCl_2$ and samples were harvested after 30 minutes. Proteins were purified on Ni^{2+}-NTA Sepharose under denaturing conditions and analyzed by Western blot using antibodies directed to Biotin and Ubiquitin respectively. H) Densitometric analysis of ubiquitin signal intensities shown in figure 4B. Ubiquitin intensities were normalized to ^{HBTH}Met30. Fold increase of ubiquitin signals were determined for each Met30 variant (n=3 independent experiments), data are represented as mean ±SD, *p* values calculated by two-tailed student's t-test: p*< 0.1, p**<0.05, p***< 0.01. I) Table showing the detected DSSO crosslinked peptides within Met30 in the absence and presence of cadmium as shown in figure 4C. Below, the total peptide count of Met30 of mass spectrometry analysis in both experimental conditions. Data shown are the combined results of three individual experiments (biological replicates).

Figure S4

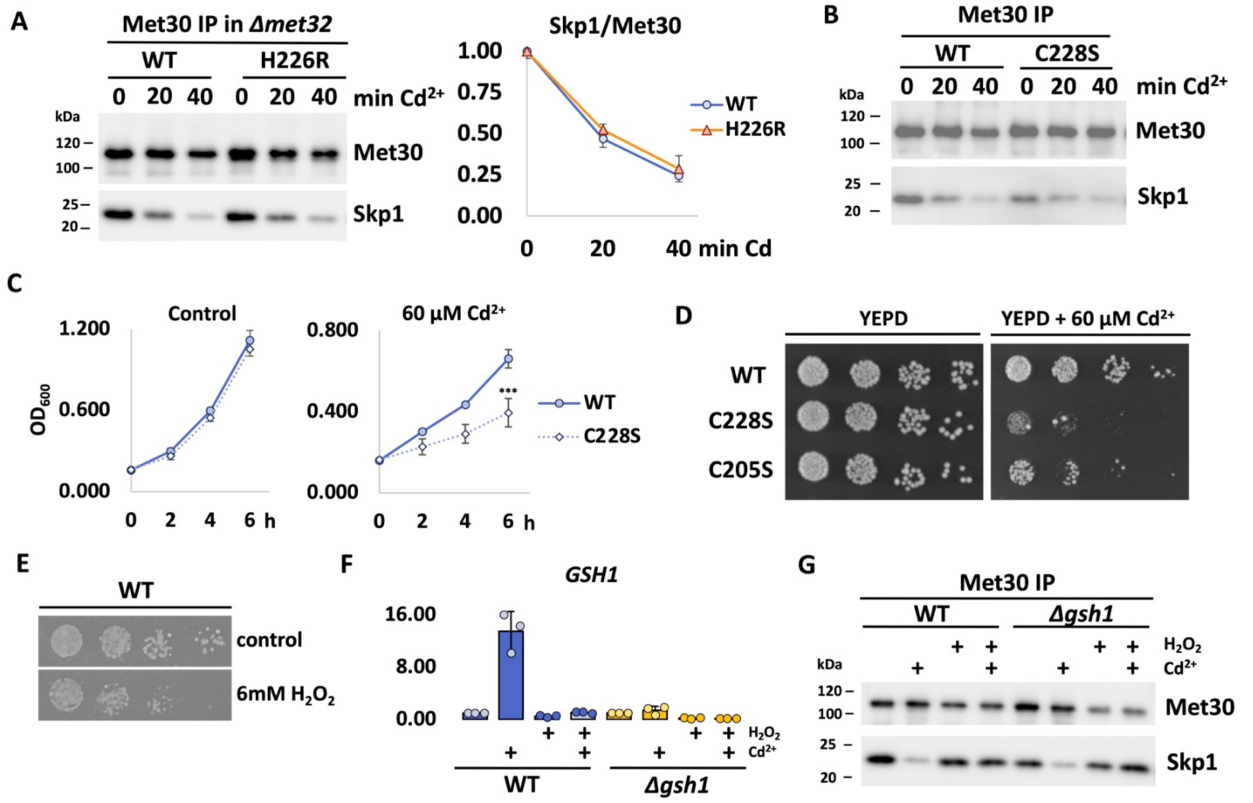

A) Dissociation kinetics of Met30 from the SCF core ligase are unaffected in H226R mutants. Cells of the *Δmet32* background were cultured at 30°C in YEPD medium and treated with 100 µM $CdCl_2$ and samples were harvested at indicated time points. ^{12xMyc}Met30 was immunoprecipitated (Met30 IP) and co-precipitated Skp1 was analyzed by Western blot. Densitometric analysis of Western blot band intensities of immunoprecipitations. For quantifications the signal intensity for WT Met30 variant at time point 0 was set to 1 and Skp1 signals were normalized to ^{12xMyc}Met30 to quantify Met30 dissociation from the core ligase (n=3). B) Dissociation kinetics of Met30 from the SCF core ligase are decreased in C228S mutants. Cells were cultured at 30°C in YEPD medium and treated with 100 µM $CdCl_2$ and samples were harvested at indicated time points. ^{12xMyc}Met30 was immunoprecipitated (Met30 IP) and co-precipitated Skp1 was analyzed by Western blot. C) C228S mutant indicates cadmium sensitivity. Cells expressing endogenous ^{12xMyc}Met30 WT or C228S respectively were cultured in YEPD medium in the absence and presence of 60µM $CdCl_2$ and samples were taken at indicated time points to measure optic density at 600 nm (n=3 independent experiments), data are represented as mean ±SD, *p* values calculated by two-tailed student's t-test: p*< 0.1, p**<0.05, p***< 0.01. D) Cells expressing ^{12Myc}Met30 WT F-box motif or C228S and C205S were cultured to logarithmic growth phase, cells were counted, and serial dilutions spotted onto YEPD plates supplemented with or without 60 µM $CdCl_2$. Plates were incubated for two days at 30°C (E) Oxidative Stress blocks cadmium induced dissociation of Met30. Cells expressing ^{12Myc}Met30 WT were cultured to logarithmic growth phase and treated with 6mM H_2O_2 for 40 minutes. Cells were then counted, and serial

dilutions spotted onto YEPD to determine survival rate after oxidative stress induction. F) Cells expressing ^{12Myc}Met30 in a wildtype background or *gsh1* knock out were cultured to logarithmic growth phase and treated with 6mM H_2O_2 for 40 minutes. Cells were then treated with 100 $CdCl_2$ and samples were harvested after 30 minutes of heavy metal exposure. RNA was extracted and expression *GSH1* was analyzed by RT-qPCR and normalized to 18S rRNA levels (n=3 independent experiments), data are represented as mean ±SD. G) ^{12xMyc}Met30 was immunoprecipitated (Met30 IP) in samples shown in Suppl. figure 4F and co-precipitated Skp1 was analyzed by Western blot.

Figure S5

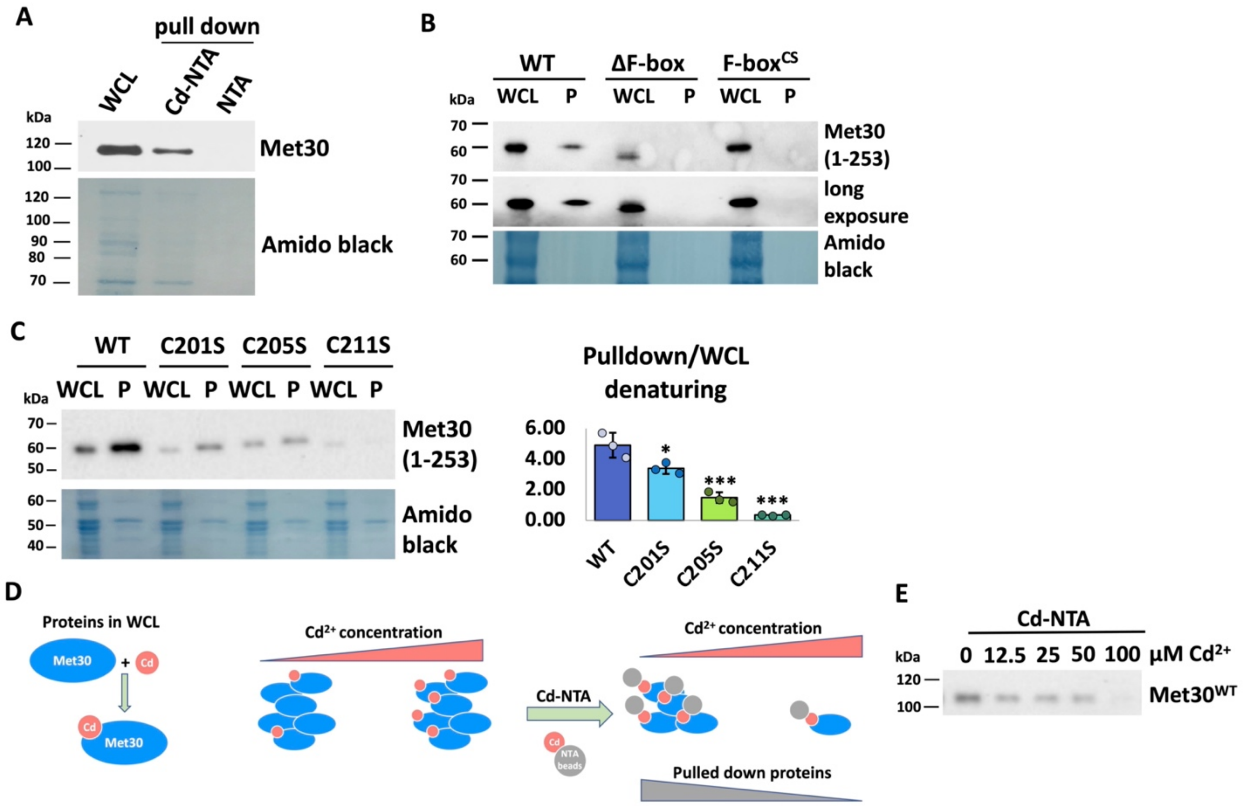

A) Met30 binds to Cd-NTA. Cells expressing endogenous ^{12xMyc}Met30 were grown in YEPD medium. A denaturing whole cell lysate was prepared and incubated with cadmium-NTA or stripped NTA respectively overnight. An equivalent of a 5x pull down was loaded and samples were analyzed by Western blot. Amido black stain shown as a loading control. B) Denaturing whole cell lysates (WCL) of cells expressing C-terminally truncated Met30 (aa 1- 253) with a WT F-box motif, F-box deletion or mutation of C201S, C205S & C211S (CS) in the F- box motif were prepared and incubated with cadmium-NTA for 90 minutes. An equivalent of a 5x pull down was loaded and the amido black stain of the membrane is shown for equal loading. C) Single cysteine mutations in F-box motif decrease cadmium binding affinity. Denaturing whole cell lysates (WCL) of cells expressing C-terminally truncated Met30 (aa 1-253) with a WT F-box motif or C201S, C205S, C211S mutation respectively were prepared and incubated with cadmium-NTA for 90 minutes. An equivalent of a 40x pull down (P) was loaded. Densitometric analysis of Western blot band intensities of Cd-NTA pull downs normalized to respective total signals (n=3 independent experiments), data are represented as mean ±SD, *p* values calculated by two-tailed student's t-test: p*< 0.1, p**<0.05, p***< 0.01. D) Cadmium chloride competes with binding to Cd-NTA. Schematic of metal competition assay. E) Native whole cell lysates of cells expressing endogenous ^{12xMyc}Met30 were prepared and incubated with indicated amounts of cadmium for 30 minutes. Extracts were incubated with Cd-NTA under denaturing conditions for 90 minutes. An equivalent of a 40x pull down (P) was loaded.

Figure S6

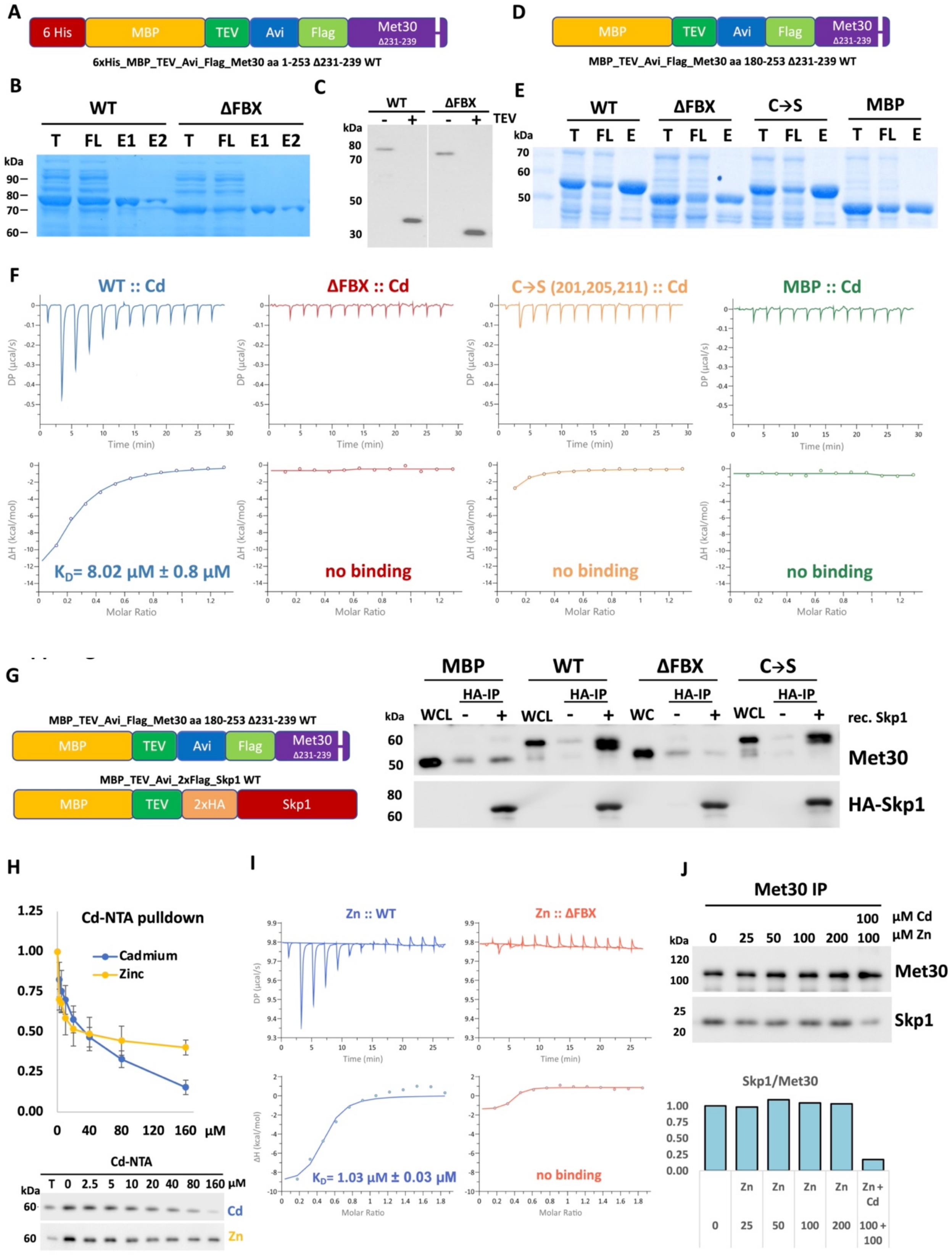
A
6 His
MBP
TEV
Avi
Flag
Met30 Δ231-239
6xHis_MBP_TEV_Avi_Flag_Met30 aa 1-253 Δ231-239 WT
D
MBP_TEV_Avi_Flag_Met30 aa 180-253 Δ231-239 WT
B
WT
ΔFBX
T FL E1 E2 T FL E1 E2
kDa
90
80
70
60
C
WT
ΔFBX
- + - + TEV
kDa
80
70
50
30
E
WT
ΔFBX
C->S
MBP
T FL E
kDa
70
60
50
F
WT :: Cd
ΔFBX :: Cd
C->S (201,205,211) :: Cd
MBP :: Cd
DP (µcal/s)
Time (min)
ΔH (kcal/mol)
Molar Ratio
KD= 8.02 µM ± 0.8 µM
no binding
no binding
no binding
G
MBP_TEV_Avi_Flag_Met30 aa 180-253 Δ231-239 WT
MBP_TEV_Avi_2xFlag_Skp1 WT
2xHA
Skp1
MBP
WT
ΔFBX
C->S
HA-IP
kDa
WCL - + WCL - + WC - + WCL - + rec. Skp1
60
50
80
60
Met30
HA-Skp1
H
Cd-NTA pulldown
Cadmium
Zinc
1.25
1.00
0.75
0.50
0.25
0.00
0 40 80 120 160 µM
Cd-NTA
kDa
T 0 2.5 5 10 20 40 80 160 µM
60 Cd
60 Zn
I
Zn :: WT
Zn :: ΔFBX
DP (µcal/s)
Time (min)
ΔH (kcal/mol)
Molar Ratio
KD= 1.03 µM ± 0.03 µM
no binding
J
Met30 IP
100 µM Cd
0 25 50 100 200 100 µM Zn
kDa
120
100
25
20
Met30
Skp1
Skp1/Met30
1.00
0.75
0.50
0.25
0.00
Zn Zn Zn Zn Zn + Cd
0 25 50 100 200 100 + 100

A) Schematic of recombinant MBP-Met30 1-253 fusion protein used for Cd-NTA pulldowns. Amino acids 231-239 were deleted for better solubility in native conditions. B) Coomassie stain of recombinant MBP-Met30 1-253 WT and ΔFBX purification. T= Total lysate, F= Flow, E1 & E2 + first and second elution, respectively. C) Purified, recombinant MBP-Met30 1-253 proteins before and after TEV cleavage. Proteins were analyzed by Western blot and proteins were detected with HRP-Streptavidin antibodies. D) Schematic of recombinant MBP-Met30 180-253 fusion protein used for ITC. E) Coomassie stain of recombinant MBP-Met30 180-253 WT, ΔFBX, C→S and MBP purification. T= Total lysate, F= Flow, E=Elution. F) Individual graphs of ITC measurement for the titration of $CdCl_2$ into recombinant MBP-TEV-Avi-Flag-Met30 variants (WT = blue, ΔFBX = red, C→S = light orange) showing a K_D of 8µM for WT Met30 and no binding for both F-Box mutants. MBP-TEV-Avi-Flag was used as a control (green). G) Truncated, recombinant Met30 WT and CS mutants form a stable complex with recombinant Skp1. Purified, recombinant MBP or MBP-Met30 containing a wt F-box motif, with the F-box motif deleted or C to S mutations were incubated with recombinant purified MBP-2xHA-Skp1 for 60 minutes. HA-Immunoprecipitation was performed and co-precipitated Met30 was analyzed by Western blot. H) Met30 exhibits high affinity towards zinc. Experiments as in panel E of figure 5, but preincubation with cadmium or zinc chloride for 30 minutes followed by binding to cadmium-NTA for 90 minutes. An equivalent of a 40x pull down (P) was loaded. Densitometric analysis of Western blot band intensities of Cd-NTA pull downs to respective total signals. **I)** ITC measurement for the titration of zinc into recombinant MBP-Met30 variants (WT = blue, ΔFBX = blood orange) showing a K_D of 1 µM for WT Met30 and no binding for the F-Box deletion mutant. Results shown are representative graphs from three independent experiments. J) Dissociation kinetics of Met30 from the SCF core ligase are unaffected by high levels of zinc *in vivo*. Cells were cultured at 30°C in YEPD medium containing depicted amounts of $ZnCl_2$ and treated with 100 µM $CdCl_2$ and samples were harvested after 30 minutes of heavy metal exposure. ^{12xMyc}Met30 was immunoprecipitated (Met30 IP) and co-precipitated Skp1 was analyzed by Western blot.

Figure S7

```
>consensus    ------LPDEVLLRIF---SFLPLRDLC-RCALVCRLWRELC-DP---------LWRR

>FBXL4        NGYFDKLPYELIQLIL---NHLTLPDLC-RLAQTCKLLSQHCCDPLQYIHLNLQPYWAKL
>FBXO11            KLPDEVVLKIF---SYLLEQDLC-RAACVCKRFSELANDPI--------LWKRK
>FBXL11                EVWMSVF---RYLSRRELC-ECMRVCKTWYKWCCDKR--------LWTKIDLSRC
>FBXL19             LPRAAWLRVF---QHLGPRELC-ICMRVCRTWSRWCYDKR--------LWPR
>FBXO45            RLPSRVLELVF---SYLELSELR-SCALVCKHWYRCLHGDE
>FBXO39       C--WAFLPDLCLCRVF---WWLGDRDRS-RAALVCRKWNQMMYSAE--------LWRYR
>FBXO22                EVVERVL---TFLPAKALL-RVACVCRLWRECV-------------RRV-----LRTH
>FBXO24           IQLFPPELVEHI---ISFLPVRDL-VALGQTCRYFHEVCDGEG-------VWRRI----CR
>FBXO34             LPHHIMVKIF---RLLPTKSLV-ALKCTCCYFKFI
>FBXO38            QLSHEVLCHIF---RYLPLQDIM-CMECLSRKLKEA
>FBXL17       D--INQLPPSILLKIF---SNLSLDERCLSASLVCKYWRDLCLDFQ--------FWKQ
>FBXO2           LDELPEPLLLRVL---AALPAAELVQACRLVCLRWKELVDGAP--------LWLLK----C
>FBXO44          INELPENILLELF---THVPARQLLLNCRLVCSLWRDLIDLVT--------LWKRK----C
>FBXO30       H--LSSLPFEVLQHIA---GFLDGFSLC-QLSCVSKLMRDVCGSL
>FBXO21          LVNLPGEVLEYIL-CCGSLTAADIG RVSSTCRRLRELCQSS
```

Alignment of mammalian F-box motifs that contain 3 or more cysteine residues. “Clustal Omega” was consulted to determine alignment (https://www.ebi.ac.uk/Tools/msa/clustalo).

Supplementary Table 1. Detailed Material Information

Chemical, Reagents and Recourses	Manufacturer	Catalog number
**Antibodies**		
Met4 (1:20.000)	Gift from M. Tyers	
Skp1(1:5000)	Gift from R. Deshaies	
Myc 9E10 (1:2000)	Santa Cruz	sc-40
HRP-Streptavidin (1:10.000)	BioLegend	405210
RGSH (1:5000)	Qiagen	34610
Goat anti mouse HRP (1:10.000)	Fisher Scientific	31430
Goat anti rabbit HRP (1:10.000)	Fisher Scientific	31460
**Bacterial strains**		
dam$^-$dcm$^-$ competent *E. coli*	NEB	C2925H
Rosetta-BL21 (DE3) E.coli	Novagen	70954-3
**Chemicals**		
Antifoam Y-30	Sigma	A5758
Cadmium chloride	Fisher Scientific	C10
Cobalt chloride hexahydrate	Sigma	C8661
Copper chloride dihydrate	EMD	CX2150-1
Dithiothreitol	Fisher Scientific	BP172
DSSO disuccinimidyl sulfoxide	Thermo Fisher	A33545
EDTA	Fisher Scientific	S311
EGTA	Fisher Scientific	E4378
Glycerol	Acros Organics	184690025
HEPES	Fisher	BP310
Leupeptin	Roche	11017101001
Pepstatin	Roche	11359053001
Phenylmethylsulfonyl floride	Sigma	P7626
SDS	Sigma	L570
Tris	Invitrogen	15505-020
Triton	Fisher Scientific	BP151-100
Trypsin (sequencing grade)	Promega	v5111
Urea	Fisher	BP169-212
**Yeast strains**		
15Daub; bar1Δ ura3Δns, ade1 his2 leu2-3112 trp1-1		Reed et al. *(49)*
PY1073; *12mycMET30::ZEO pep4::URA3*		Yen et al. *(15)*
PY2331; *CDC48RGS6xHis::KAN 12MycMET30_wt::ZEO Shp1-3xHA::TRP pep4::URA*		Lauinger et al. *(28)*
PY2340;*CDC48RGS6xHis::KAN12MycMET30_C201SC205SC211S::ZEO Shp1-3xHA::TRP pep4::URA*		This study
PY2341; *CDC48RGS6xHis::KAN 12MycMET30_C201S::ZEO Shp1-3xHA::TRP pep4::URA*		This study
PY2342;*CDC48RGS6xHis::KAN 12MycMET30_C205S::ZEO Shp1-3xHA::TRP pep4::URA*		This study
PY2343; *CDC48RGS6xHis::KAN 12MycMET30_C211S::ZEO Shp1-3xHA::TRP pep4::URA*		This study

PY2344; *12mycMET30_Δ95-99::ZEO pep4::URA3*		This study
PY2345; *12mycMET30_Δ236-239::ZEO pep4::URA4*		This study
PY1310; *met32::HYG met30::KAN pep4::URA*		Kaiser et al. *(19)*
PY2346*met32::HYG met30::KAN pep4::URA pYLEU_met30_12xMYC_Met30 wt*		This study
PY2347*met32::HYG met30::KAN pep4::URA pYLEU_met30_12xMYC_Met30 Δ1-113*		This study
PY2348*met32::HYG met30::KAN pep4::URA pYLEU_met30_12xMYC_Met30 Δ113-180*		This study
PY2349*met32::HYG met30::KAN pep4::URA pYLEU_met30_12xMYC_Met30 1-253*		This study
PY2350*met32::HYG met30::KAN pep4::URA pYLEU_met30_12xMYC_Met30 with Mfb1 F-Box*		This study
PY2351*met32::HYG met30::KAN pep4::URA pYLEU_met30_12xMYC_Met30 with Grr1-Box*		This study
PY2352*met32::HYG met30::KAN pep4::URA pYLEU_met30_12xMYC_Met30 with Cdc4-Box*		This study
PY2353*met32::HYG met30::KAN pep4::URA pYLEU_met30_12xMYC_Met30 1-253 C201S*		This study
PY2354*met32::HYG met30::KAN pep4::URA pYLEU_met30_12xMYC_Met30 1-253 C205S*		This study
PY2355*met32::HYG met30::KAN pep4::URA pYLEU_met30_12xMYC_Met30 1-253 C211S*		This study
PY2356; *CDC48RGS6xHis::KAN 12MycMET30_C228S::ZEO Shp1-3xHA::TRP pep4::URA*		This study
PY2357; *met32::galTRP 12MycMET30_H226R::ZEO pep4::URA*		This study
PY2358 *met32::HYG met30::KAN pep4::URA pYLEU_met30_12xMYC_Met30 1-253 C228S*		This study
**Oligonucleotides**		
MET25F: GCCACCACTTCTTATGTTTTCG		
MET25R: AGCAGCAGCACCACCTTC		
GSH1F: TGACAGCATCCATCAGGACCAG		
GSH1R: GGAAGCCAGTTTCGCCTCTTTG		
18SrRNAF: GTGGTGCTAGCATTTGCTGGTTAT		
18SrRNAR: CGCTTACTAGGAATTCCTCGTTGAA		
016Met30d1-113XhoF tttCTCGAGCATGCATCTAGAGGGCCCGATTTGAAACGAACGCAA GAGATTAATGC		
017Met30utrSmaIR aaaCCCGGGaggcatgctcataccagcaaaag		
028Met30ATGXhoF tttCTCGAGCATGCATCTAGAGGGCCCATGAGGAGAGAGAGGCA AAGG		
030Met30 253 R MscSma aaaCCCGGGTGGCCActaTATCCGCGCACGTTTCATG		
031Met30d113NotR AAAtgcggccgcGTAGCACGCTGTATGAGTTGG		
032Met30d113145F tttgcggccgcaAGCAATTCCAATGACAAGATACG		

034Met30d113180F<br>tttgcggccgcaATCGACTTCATCAGCATTCTGC		
173Met30g620F<br>GAAAGATAAATGATCACCACGAGAGTGTGCCGCAAGGTTTTAGA<br>GCTAGAAATAGCAAGTTAAAA		
174Met30g620R<br>TTTTAACTTGCTATTTCTAGCTCTAAAACCTTGCGGCACACTCTCG<br>TGGTGATCATTTATCTTTC		
201Mfb1FBXF<br>CGTCACTCGTTACACACATGATCAAGGAAAGGTCCCTGACAAACT<br>TA		
202Mfb1FBXR<br>CTGTCTATGTGCTGCTCGCAAGCATTTCTGTAAACAATGTTCTC		
205Mfb1CtagF<br>TGTAATCAAACGGCTTGACGCTAATACCGATTTTAATATA CGG<br>ATC CCC GGG TTA ATT AA		
206Mfb1CtagR<br>GAAAACTCTTCCAAGCAAAGTCGGTTTGAGGCGTTTTCTGGAATT<br>CGAGCTCGTTTAAAC		
263Met30g680F<br>GAAAGATAAATGATCATGTGCGAGCAGCACATAGACGTTTTAGA<br>GCTAGAAATAGCAAGTTAAAA		
264Met30g680R<br>TTTTAACTTGCTATTTCTAGCTCTAAAACGTCTATGTGCTGCTCGC<br>ACATGATCATTTATCTTTC		
282M30FBXC201SF<br>CTTGAGTTATCTGGATTcCCAATCTCTTTGCAACGCtACtAGgGTGT<br>GCCGCAAaTGGCAGAAGCTCGCGGATGACGACAGGGTATGGTA<br>CC		
283M30FBXC201SR<br>GGTACCATACCCTGTCGTCATCCGCGAGCTTCTGCCAtTTGCGGC<br>ACACcCTaGTaGCGTTGCAAAGAGATTGGgAATCCAGATAACTCA<br>AG		
284M30FBXS205SF<br>CTTGAGTTATCTGGATTGCCAATCTCTTTcCAACGCtACtAGgGTGT<br>GCCGCAAaTGGCAGAAGCTCGCGGATGACGACAGGGTATGGTA<br>CC		
285M30FBXS205SR<br>GGTACCATACCCTGTCGTCATCCGCGAGCTTCTGCCAtTTGCGGC<br>ACACcCTaGTaGCGTTGgAAAGAGATTGGCAATCCAGATAACTCA<br>AG		
286M30FBXC211SF<br>CTTGAGTTATCTGGATTGCCAATCTCTTTGCAACGCtACtAGgGTG<br>TcCCGCAAaTGGCAGAAGCTCGCGGATGACGACAGGGTATGGTA<br>CC		
287M30FBXC211SR<br>GGTACCATACCCTGTCGTCATCCGCGAGCTTCTGCCAtTTGCGGg<br>ACACcCTaGTaGCGTTGCAAAGAGATTGGCAATCCAGATAACTCA<br>AG		

290M30C236C239SF GATGACGACAGGGTATGGTACCACATGTGCGAGCAGCACATAG ACAGaAAATcTCCCAACTcTGGCTGGGGGCTGCCTCTTTTGCACAT G		
291M30C236C239SR CATGTGCAAAAGAGGCAGCCCCCAGCCAgAGTTGGGAgATTTtCT GTCTATGTGCTGCTCGCACATGTGGTACCATACCCTGTCGTCATC		
292M30d236-239toAAA GATGACGACAGGGTATGGTACCACATGTGCGAGCAGCACATAG ACAGaAAAgcggccgctGGCTGGGGGCTGCCTCTTTTGCACATG		
293M30d236-239toAAA CATGTGCAAAAGAGGCAGCCCCCAGCCagcggccgcTTTtCTGTCTA TGTGCTGCTCGCACATGTGGTACCATACCCTGTCGTCATC		
310 AviFlagM30F: tggccaGGTCTGAACGACATCTTCGAGGCTCAGAAAATCGAATGG CACGAAggaggtGACTACAAAGACGATGATGACAAAggcgcgcctAG GAGAGAGAGGCAAAGGAT		
311 M30-253R CCGGATCgcggccgcCTATATCCGCGCACGTTTCATG		
312 pET28F AGgcggccgcGATCCGGCTGCTAACAAAG		
313 pET28R GTTCAGACCtggccaATGAGTAATATCACCTTGAAAATAAAGATT		
314M30d231-239PacF gccttaattaaaGGCTGGGGGCTGCCTCTTTT		
315M30d231-239wtR GCCtttaattaaggcCTGCTCGCACATGTGGTACCA		
316M30d231-239dfbxR GCCtttaattaaggcCTGCTCGCAGTCTAGAATGCTGATG		
**Plasmids**		
pML107 pSNR52sgRNA casette CAS9-gWY001 casette LEU2		Laughery et al. *(50)*
pML107 g620Met30 pSNR52sgRNA casette CAS9-gWY001 casette LEU2 + Met30g620		This study
pML107 g680Met30 pSNR52sgRNA casette CAS9-gWY001 casette LEU2 + Met30g680		This study
pYLEU_met30_12xMYC_Met30 wt		This study
pYLEU_met30_12xMYC_Met30 ΔFBX (pPK680)		
pYLEU_met30_12xMYC_Met30 Δ1-113		This study
pYLEU_met30_12xMYC_Met30 Δ113-180		This study
pYLEU_met30_12xMYC_Met30 FBX of Cdc4		This study
pYLEU_met30_12xMYC_Met30 FBX of Grr1		This study
pYLEU_met30_12xMYC_Met30 FBX of Mfb1		This study
pYLEU_met30_12xMYC_Met30 1-253		This study
pYLEU_met30_12xMYC_Met30 1-253ΔFBX		This study
pYLEU_met30_12xMYC_Met30 1-253 C201S		This study
pYLEU_met30_12xMYC_Met30 1-253 C205S		This study
pYLEU_met30_12xMYC_Met30 1-253 C211S		This study
pYLEU_met30_12xMYC_Met30 1-253 C228S		This study
pET28-6His-MBP-TEV	Addgene	69929
pET28-6His-MBP-TEV-Avi-Flag-Met30_1-253wt (Δ231-239)		This study

pET28-6His-MBP-TEV-Avi-Flag-Met30_1-253ΔFBX (Δ231-239)		This study
pET28-MBP-TEV-Avi-Flag		This study
pET28-MBP-TEV-Avi-Flag-Met30_180-253wt (Δ231-239)		This study
pET28-MBP-TEV-Avi-Flag-Met30_180-253ΔFBX (Δ231-239)		This study
pET28-MBP-TEV-Avi-Flag-Met30_180-253 C→S (201,205,211) (Δ231-239)		This study
**Software**		
ImageJ	(Schneider et al., 2012)	https://imagej.nih.gov/ij/
Image Lab	Biorad	
MicroCal PEAQ-ITC Analysis Software v1.41	Malvern	
ProteinProspector (v.6.3.3)		
MSConvert (ProteoWizard 3.0.10738)		
**Other**		
Ni-NTA agarose	Qiagen	30210
Myc-trap	Chromotek	yta-100
Streptavidin Resin	Thermo Fisher	20349
Amylose resin	NEB	E8021S
polyvinylidene difluoride membrane Immobilon-P	Merck	IVPH00010
MP FastPrep 24	MP	
RNeasy Plus Mini Kit	Qiagen	
Super Sript II Reverse Transcriptase	Invitrogen	
CFX Connect RT-PCR machine	Biorad	
iTaq Universal SYBR Green SuperMix	Biorad	
Micro-Cal PEAQ-ITC system	Malvern	
