# Supplementary material for: Cadmium binding by the F-box domain induces p97-mediated SCF complex disassembly to activate stress response programs
Source: Nat Commun. 2024 May 8;15:3894. doi: 10.1038/s41467-024-48184-6 (PMC11079001; doi:10.1038/s41467-024-48184-6)
Supplement: Supplementary file 3 — Description of Additional Supplementary Files [file 41467_2024_48184_MOESM3_ESM.pdf]

File name: Supplementary Data 1
Description: Lauinger LUMOS DSSO Met30WT Cntrl vs. Cadmium.
Excel file summarizes most important information from MS runs of the 3 individual experiments. File provides information about identified proteins, their peptide count, coverage as well as intrasubunit interlinks and more.
