# Supplementary material for: Cadmium binding by the F-box domain induces p97-mediated SCF complex disassembly to activate stress response programs
Source: Nat Commun. 2024 May 8;15:3894. doi: 10.1038/s41467-024-48184-6 (PMC11079001; doi:10.1038/s41467-024-48184-6)

Figure 1

B

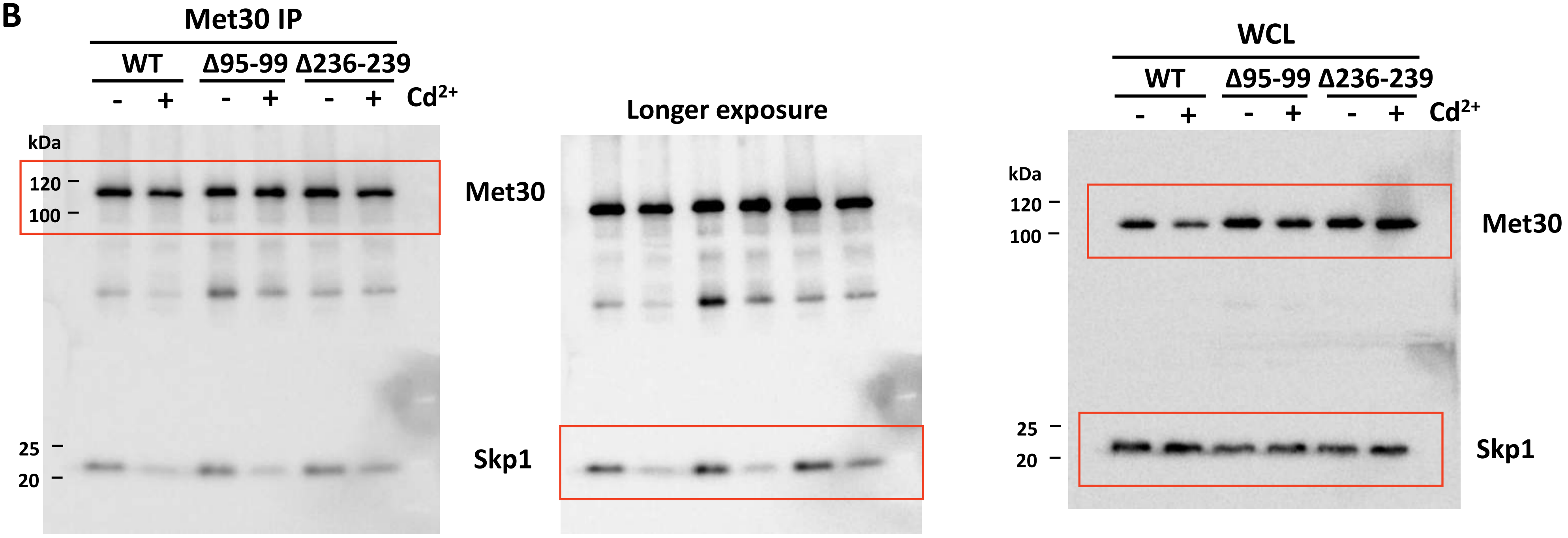

Met30 IP
WT
Δ95-99
Δ236-239
- + - + - + Cd2+
kDa
120
100
25
20
Met30
Skp1
Longer exposure
WCL
WT
Δ95-99
Δ236-239
- + - + - + Cd2+
kDa
120
100
25
20
Met30
Skp1

Figure 1

D

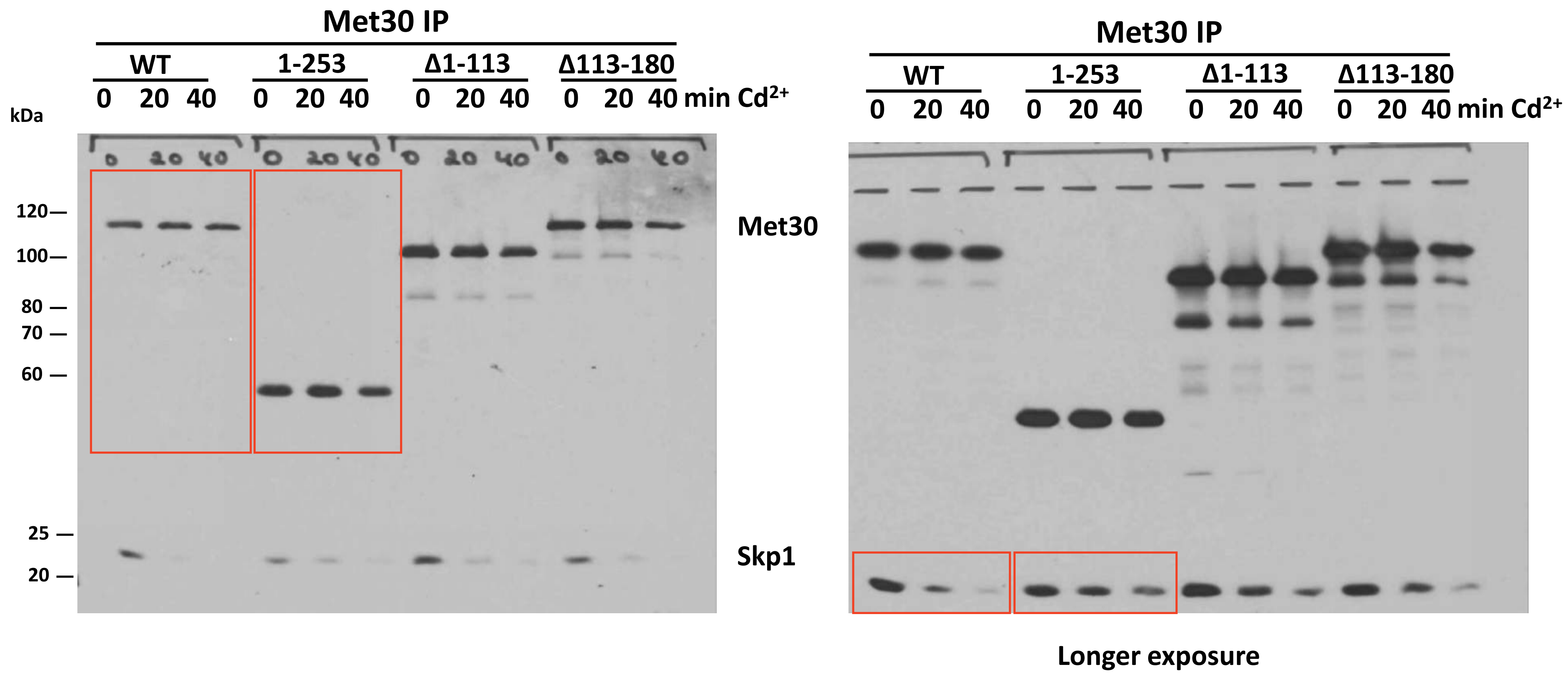
Met30 IP
WT
1-253
Δ1-113
Δ113-180
0 20 40 min Cd2+
kDa
120
100
80
70
60
25
20
Met30
Skp1
Met30 IP
WT
1-253
Δ1-113
Δ113-180
0 20 40 min Cd2+
Longer exposure

Figure 1

D

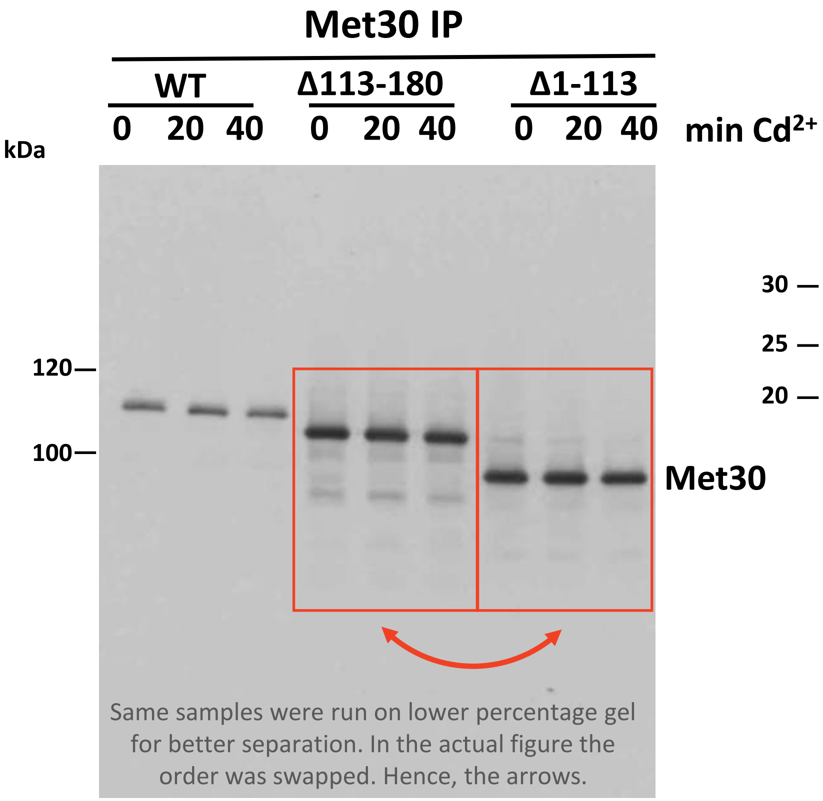

Met30 IP
WT
Δ113-180
Δ1-113
0 20 40 0 20 40 0 20 40 min Cd2+
kDa
120—
100—
Met30
Same samples were run on lower percentage gel for better separation. In the actual figure the order was swapped. Hence, the arrows.

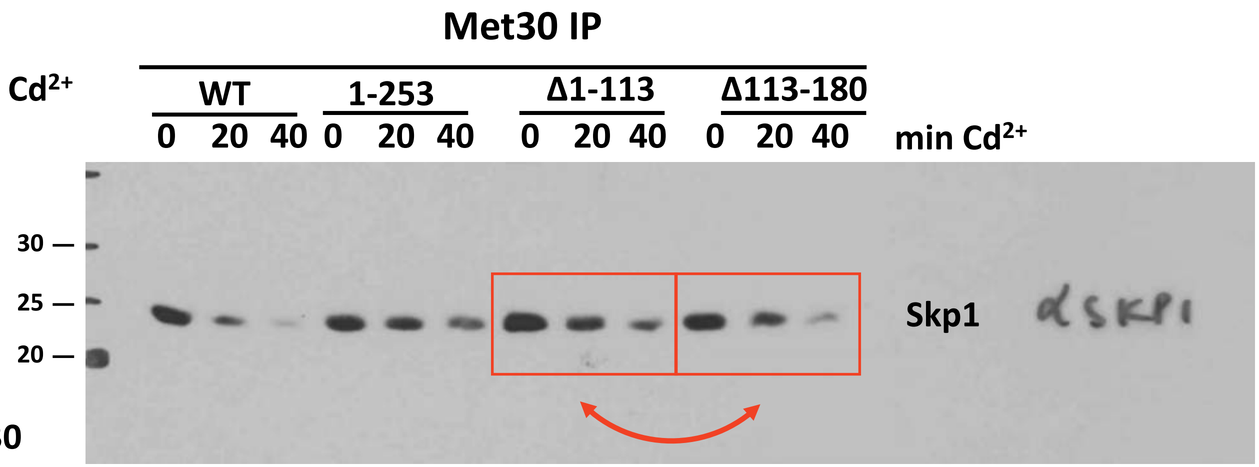

Met30 IP
WT
1-253
Δ1-113
Δ113-180
0 20 40 0 20 40 0 20 40 0 20 40 min Cd2+
30 —
25 —
20 —
Skp1

Figure 2

C

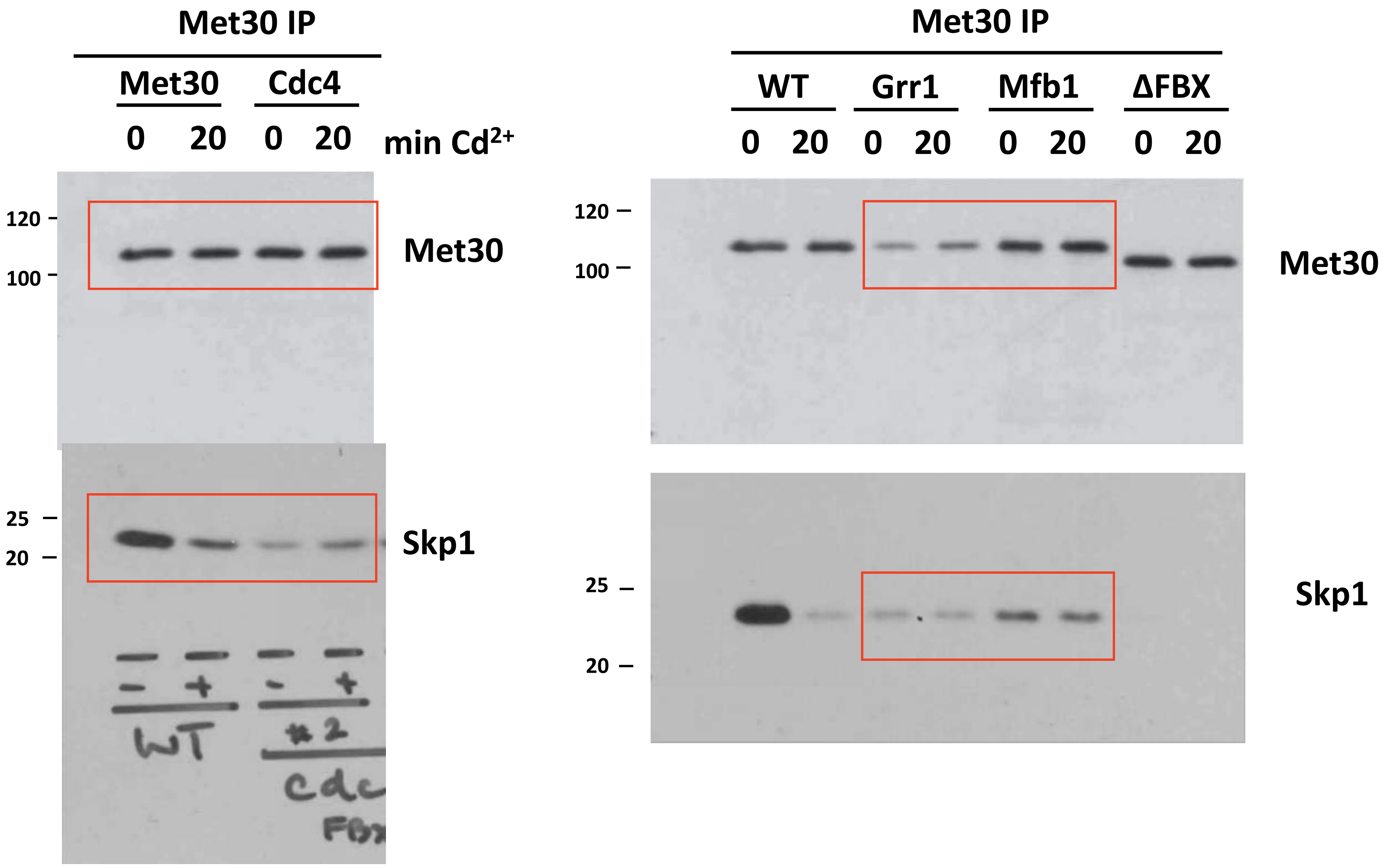

Met30 IP
Met30
Cdc4
0
20
0
20
min Cd2+
120
100
Met30
25
20
Skp1
WT
Met30 IP
WT
Grr1
Mfb1
ΔFBX
0
20
0
20
0
20
0
20
120
100
Met30
25
20
Skp1

Figure 2

D

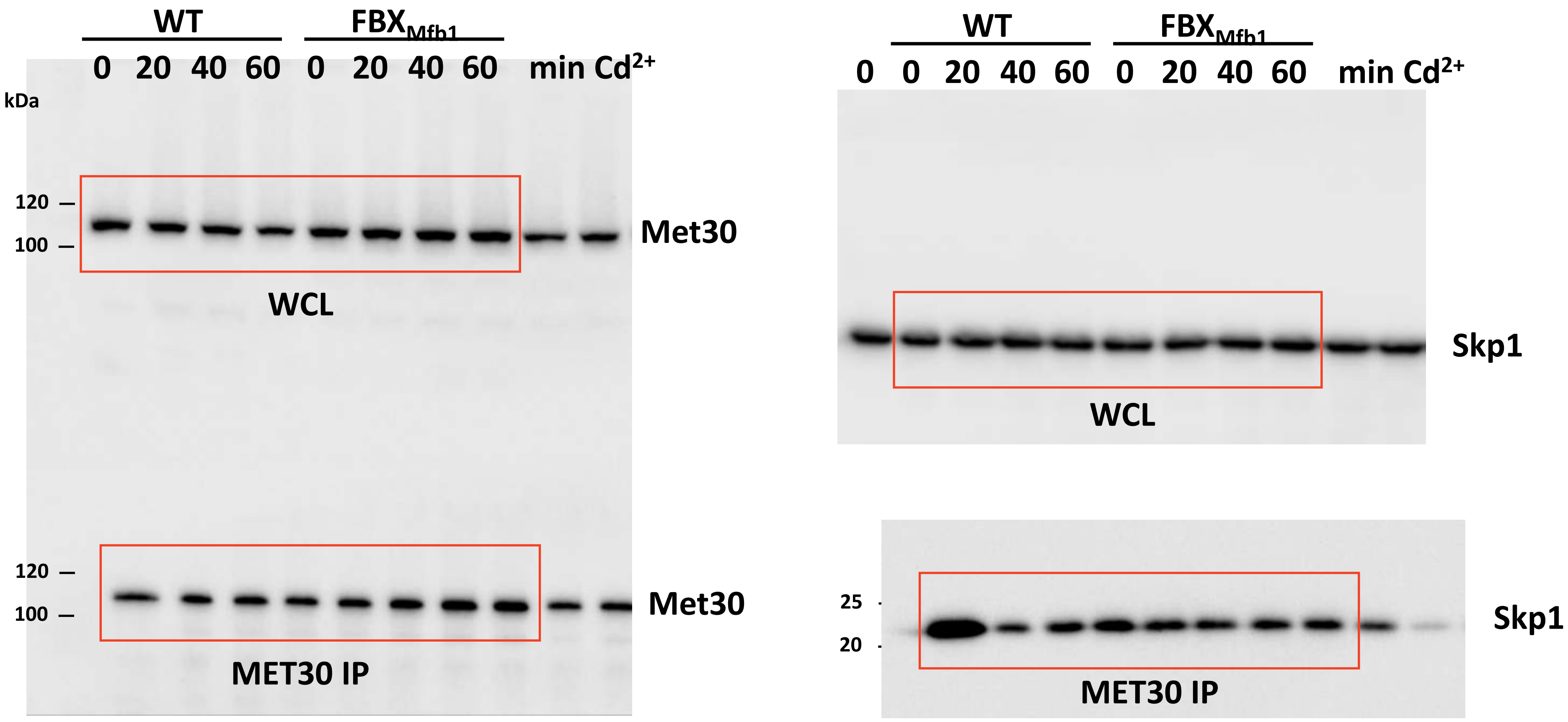

WT
FBX$_{Mfb1}$
0 20 40 60 0 20 40 60 min Cd^{2+}
kDa
120
100
Met30
WCL
120
100
Met30
MET30 IP
WT
FBX$_{Mfb1}$
0 0 20 40 60 0 20 40 60 min Cd^{2+}
Skp1
WCL
25
20
Skp1
MET30 IP

Figure 3

A

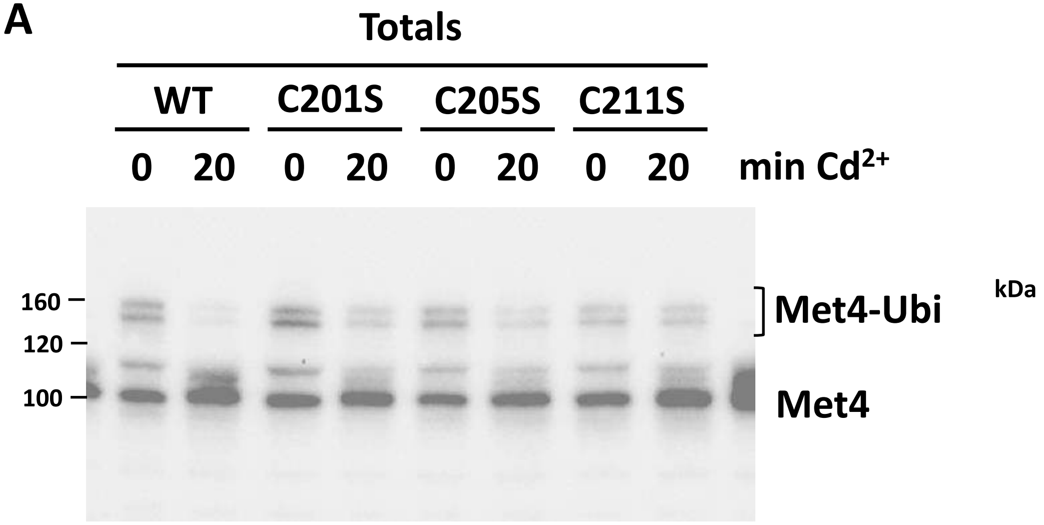
Totals
WT
C201S
C205S
C211S
0 20 0 20 0 20 0 20 min Cd2+
160
120
100
Met4-Ubi
Met4
kDa

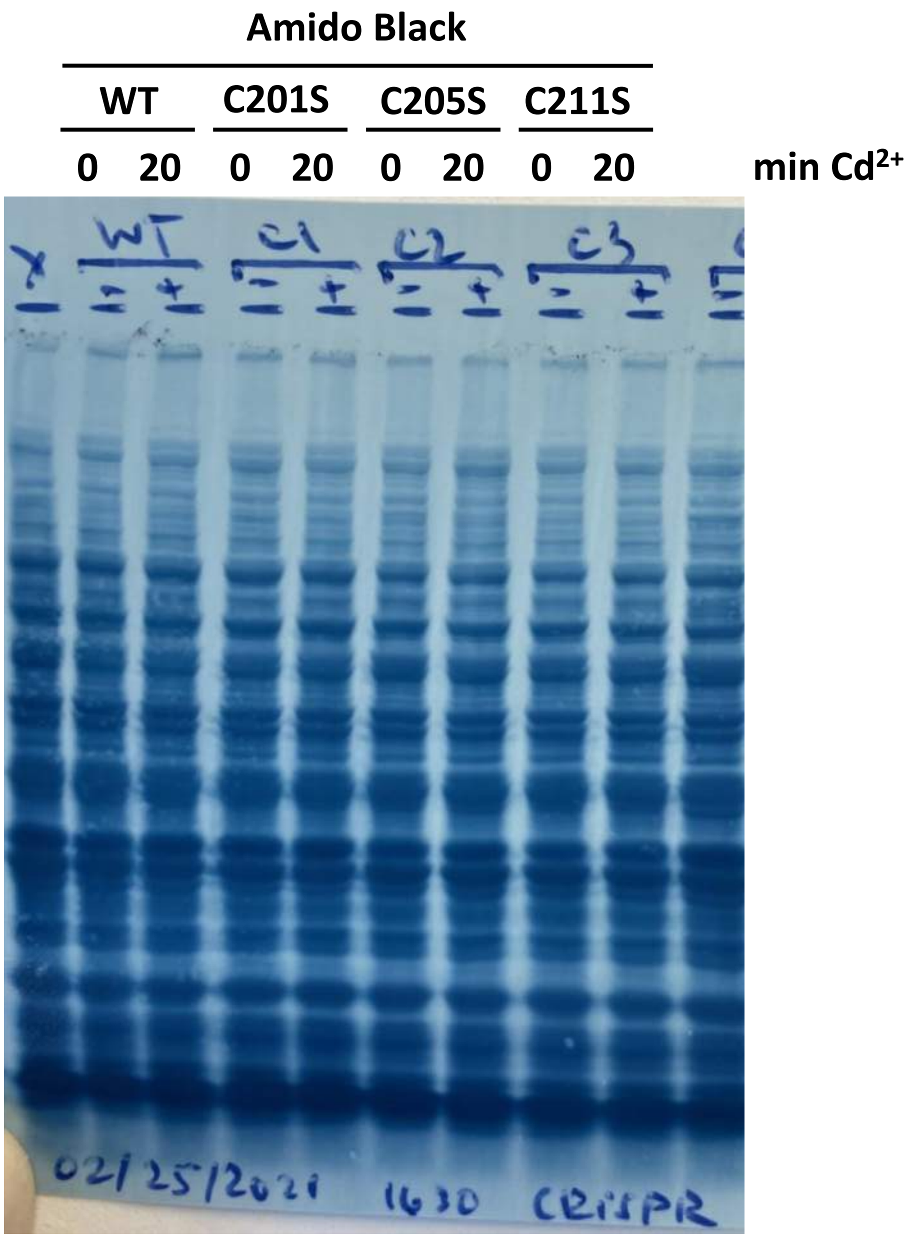
Amido Black
WT
C201S
C205S
C211S
0 20 0 20 0 20 0 20 min Cd2+
WT
C1
C2
C3
02/25/2021
1630
CRISPR

Figure 3
B

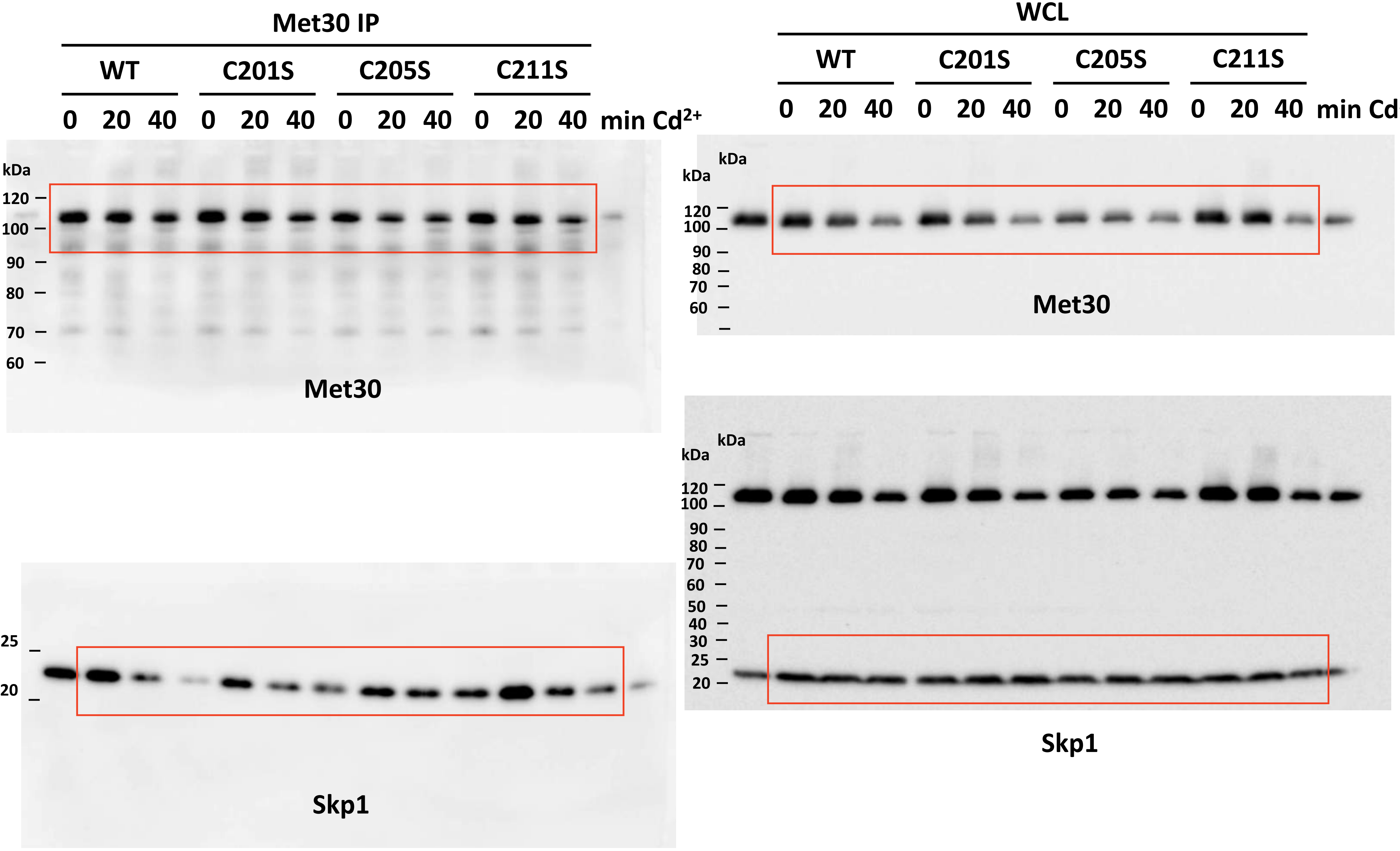

Met30 IP
WT
C201S
C205S
C211S
0 20 40 0 20 40 0 20 40 0 20 40 min Cd2+
kDa
120
100
90
80
70
60
Met30
25
20
Skp1
WCL
WT
C201S
C205S
C211S
0 20 40 0 20 40 0 20 40 0 20 40 min Cd
kDa
120
100
90
80
70
60
Met30
kDa
120
100
90
80
70
60
50
40
30
25
20
Skp1

Figure 4

A

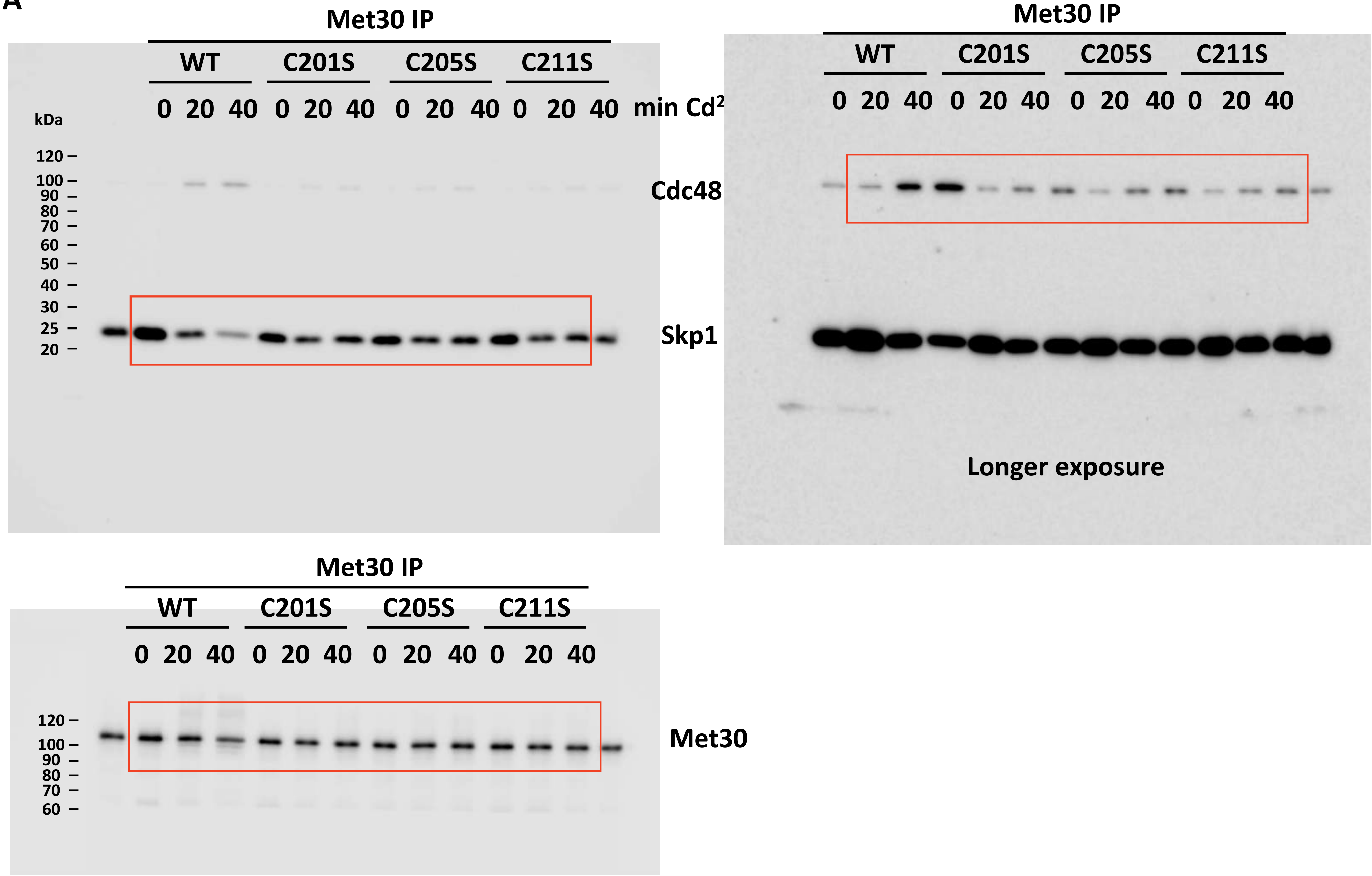

Met30 IP
WT
C201S
C205S
C211S
0 20 40 0 20 40 0 20 40 0 20 40 min Cd²
kDa
120
100
90
80
70
60
50
40
30
25
20
Cdc48
Skp1
Met30 IP
WT
C201S
C205S
C211S
0 20 40 0 20 40 0 20 40 0 20 40
Longer exposure
Met30 IP
WT
C201S
C205S
C211S
0 20 40 0 20 40 0 20 40 0 20 40
120
100
90
80
70
60
Met30

Figure 4

B

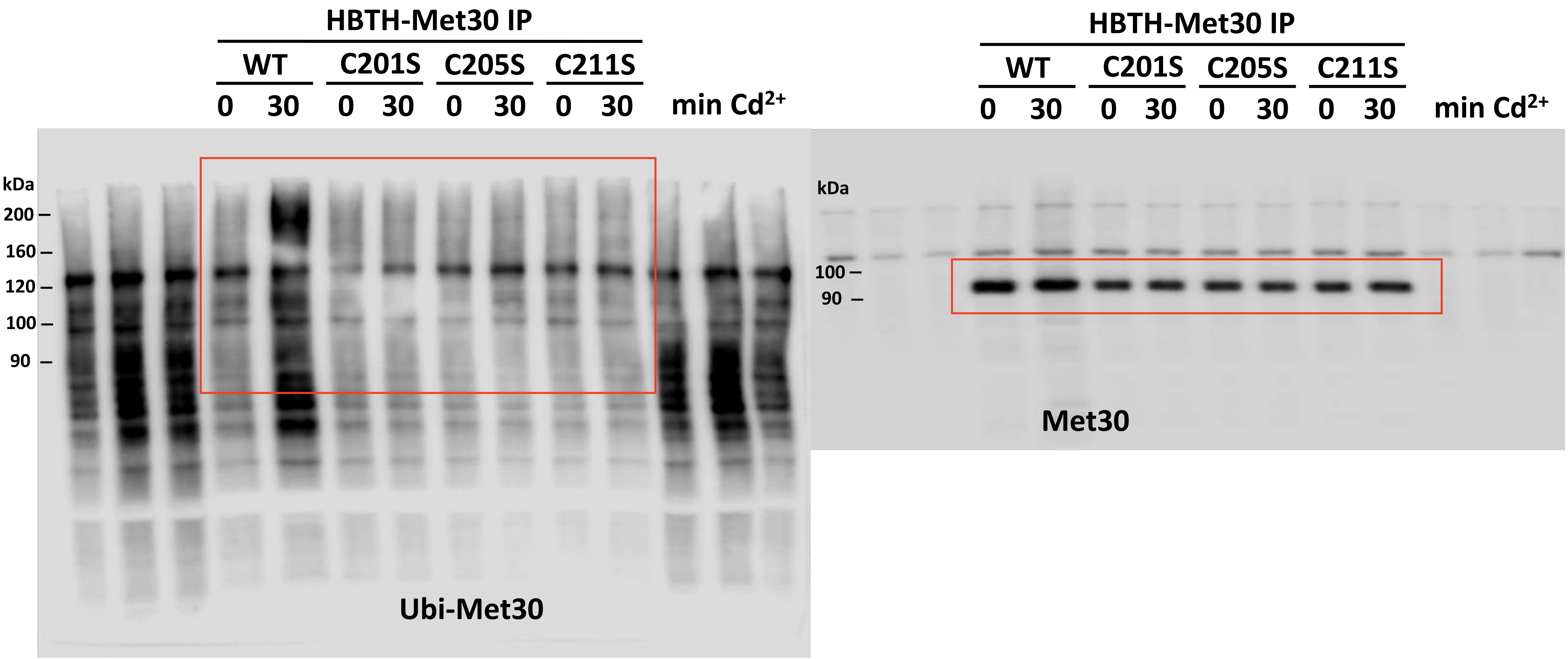
HBTH-Met30 IP
WT
C201S
C205S
C211S
0 30 0 30 0 30 0 30 min Cd2+
kDa
200
160
120
100
90
Ubi-Met30
HBTH-Met30 IP
WT
C201S
C205S
C211S
0 30 0 30 0 30 0 30 min Cd2+
kDa
100
90
Met30

Figure 4

D

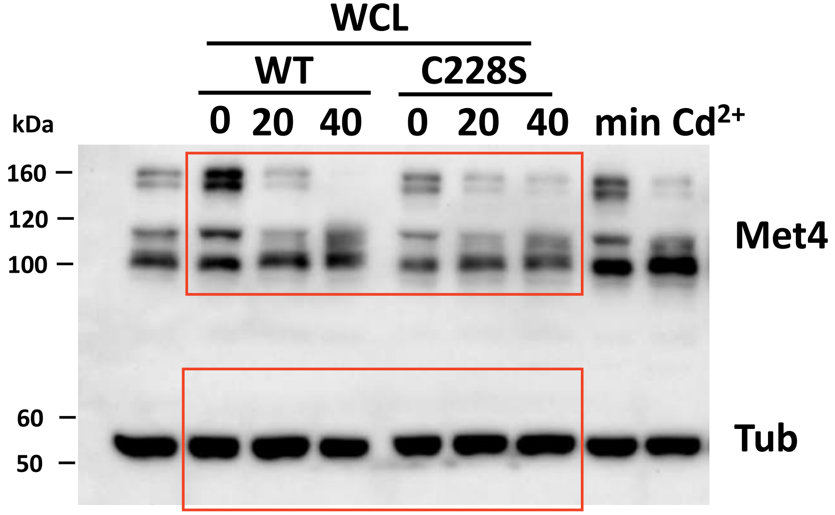

Figure 5

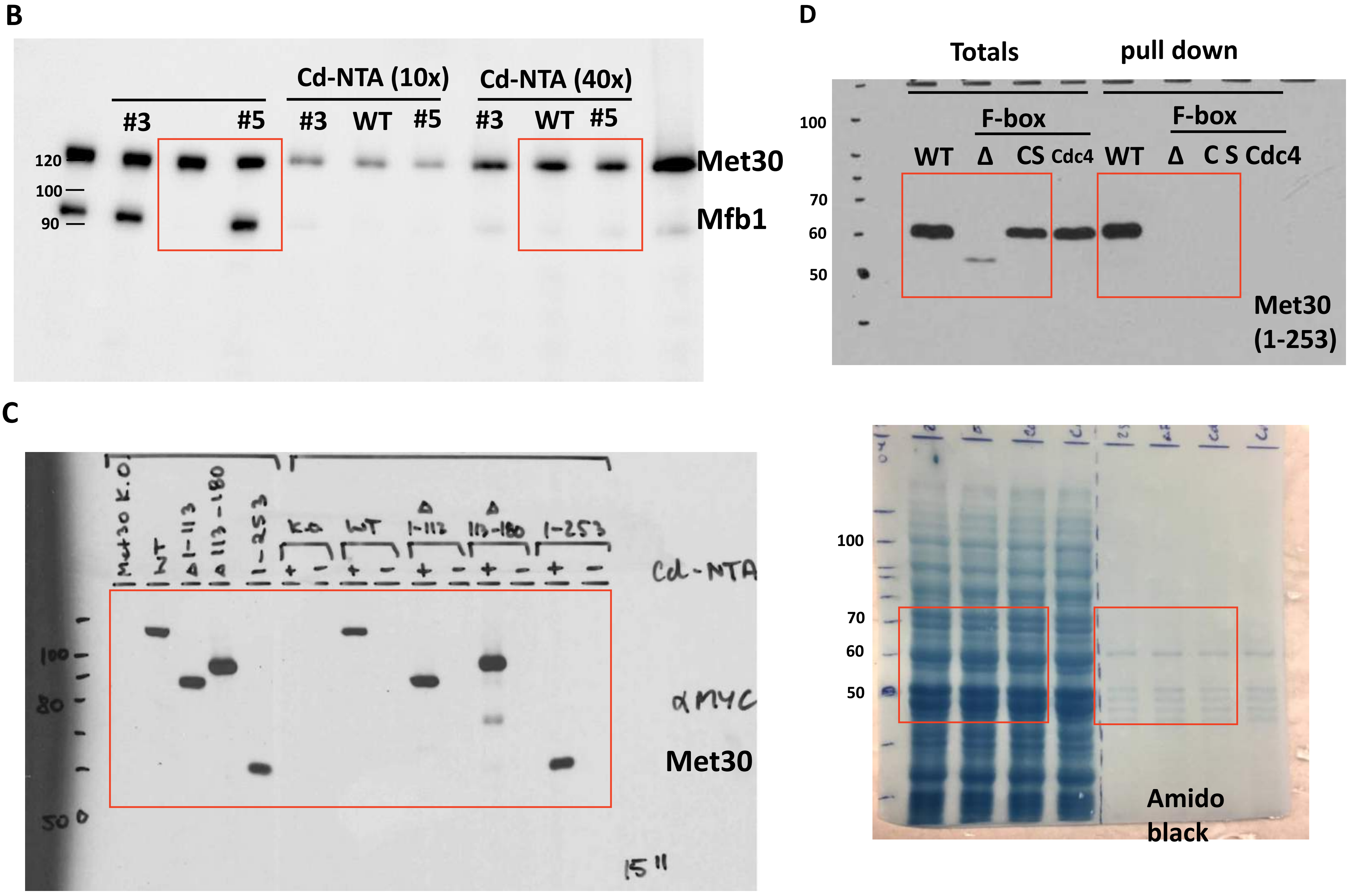
B
Cd-NTA (10x)
Cd-NTA (40x)
#3
#5
#3
WT
#5
#3
WT
#5
120
100
90
Met30
Mfb1
C
Cd-NTA
αMYC
Met30
D
Totals
pull down
F-box
F-box
WT
Δ
CS
Cdc4
WT
Δ
C S
Cdc4
100
70
60
50
Met30
(1-253)
100
70
60
50
Amido
black

Figure 5

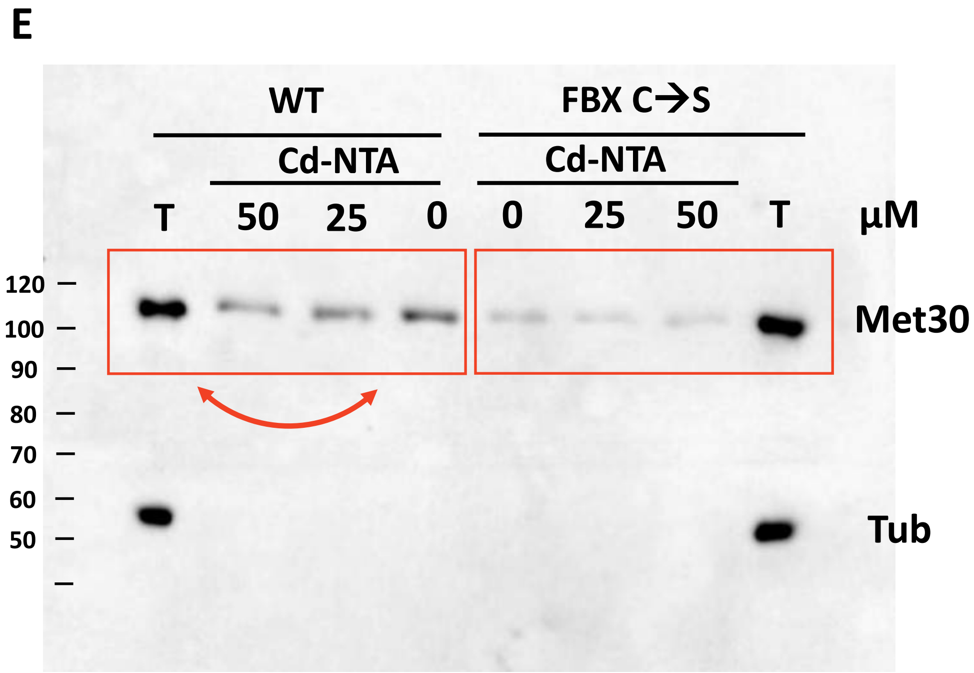

E
WT
FBX C→S
Cd-NTA
Cd-NTA
T 50 25 0 0 25 50 T µM
120
100
90
80
70
60
50
Met30
Tub

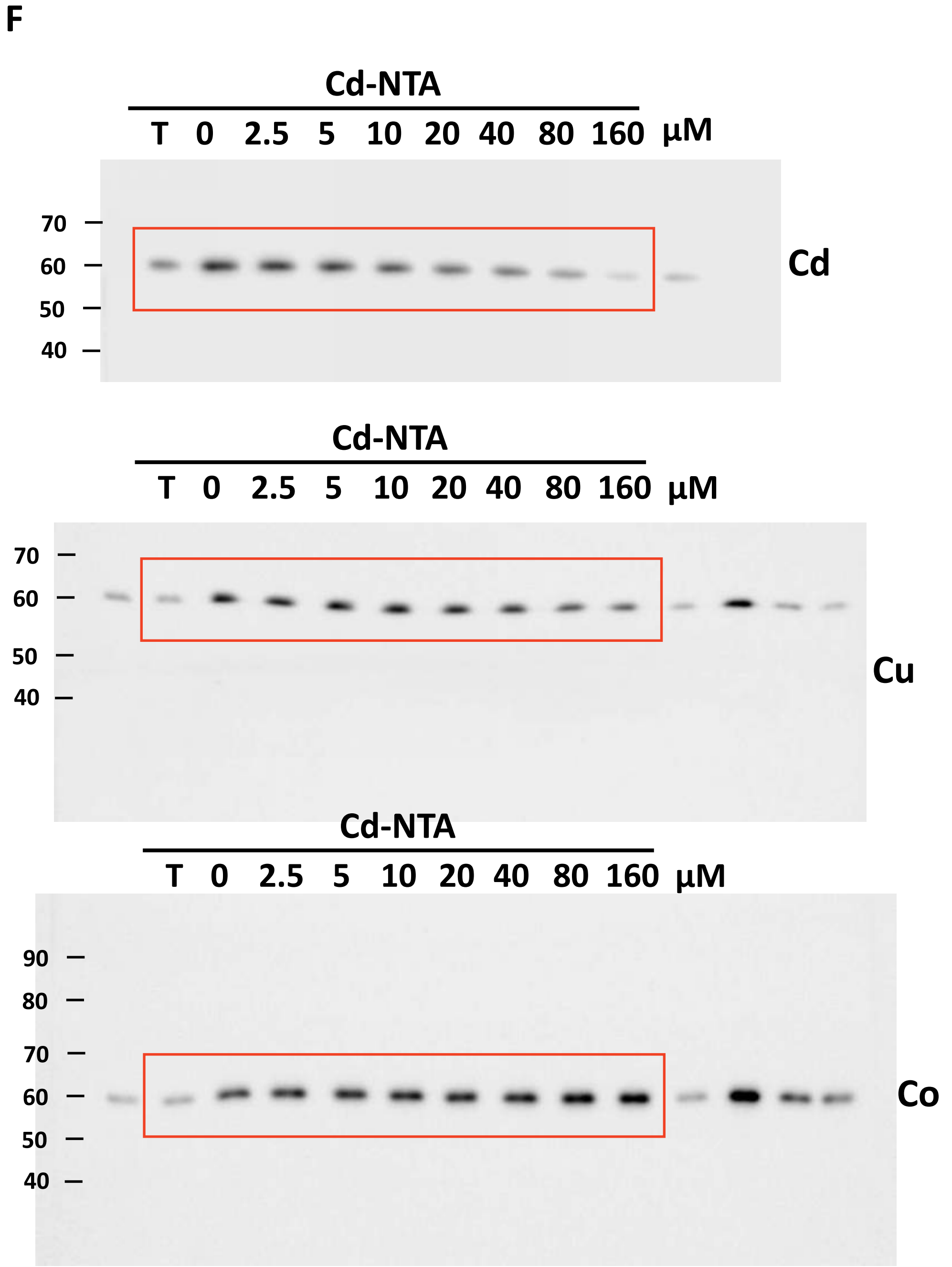

F
Cd-NTA
T 0 2.5 5 10 20 40 80 160 µM
70
60
50
40
Cd
Cd-NTA
T 0 2.5 5 10 20 40 80 160 µM
70
60
50
40
Cu
Cd-NTA
T 0 2.5 5 10 20 40 80 160 µM
90
80
70
60
50
40
Co

Figure 6

A

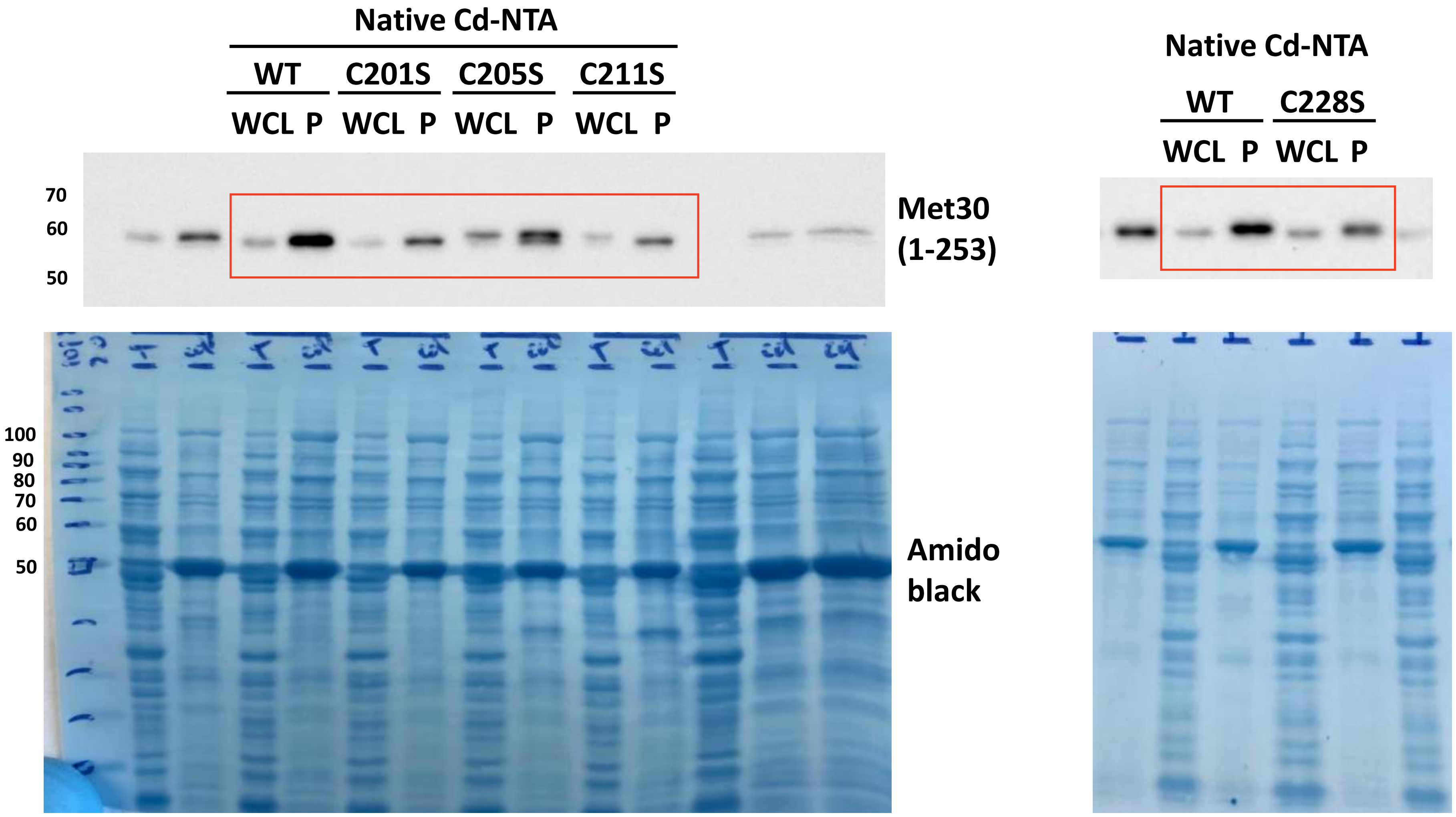
Native Cd-NTA
WT
C201S
C205S
C211S
WCL
P
WCL
P
WCL
P
WCL
P
70
60
50
Met30
(1-253)
Native Cd-NTA
WT
C228S
WCL
P
WCL
P
100
90
80
70
60
50
Amido
black

Figure 6

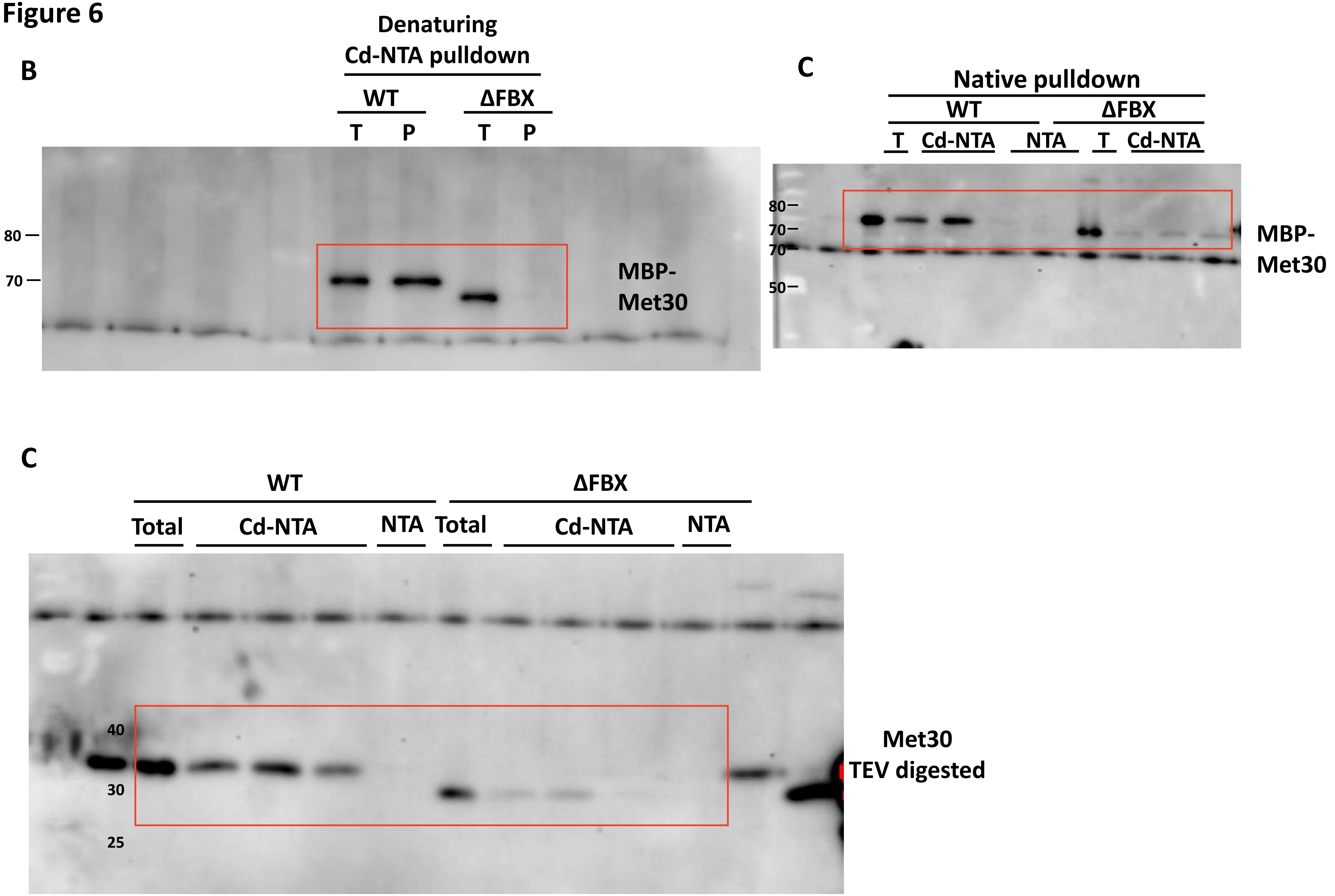
B
Denaturing
Cd-NTA pulldown
WT
ΔFBX
T
P
T
P
80
70
MBP-
Met30
C
Native pulldown
WT
ΔFBX
T
Cd-NTA
NTA
T
Cd-NTA
80
70
70
50
MBP-
Met30
C
WT
ΔFBX
Total
Cd-NTA
NTA
Total
Cd-NTA
NTA
40
30
25
Met30
TEV digested

Suppl. Figure 1

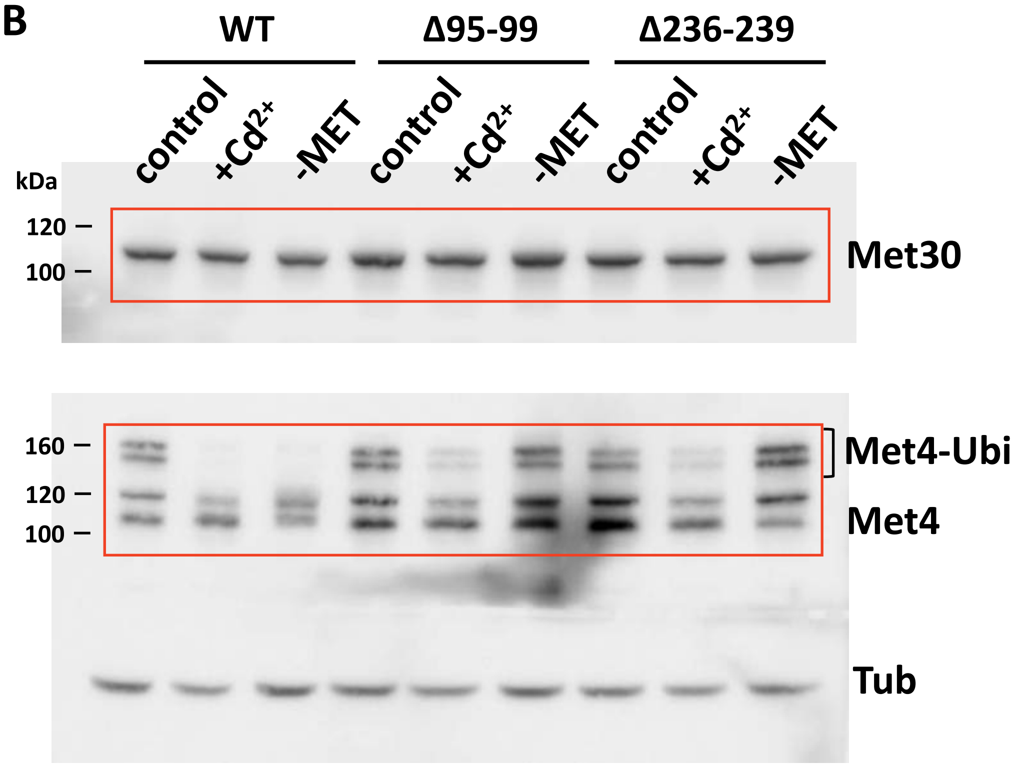

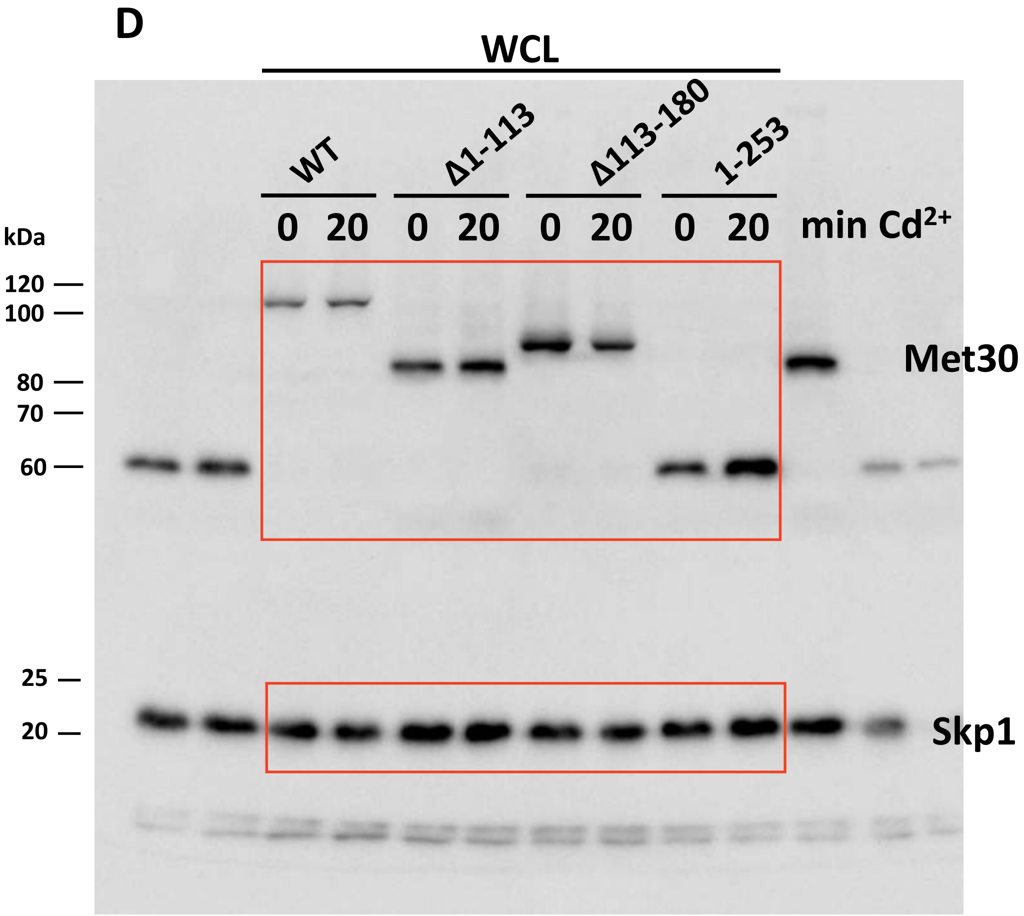

Suppl. Figure 2

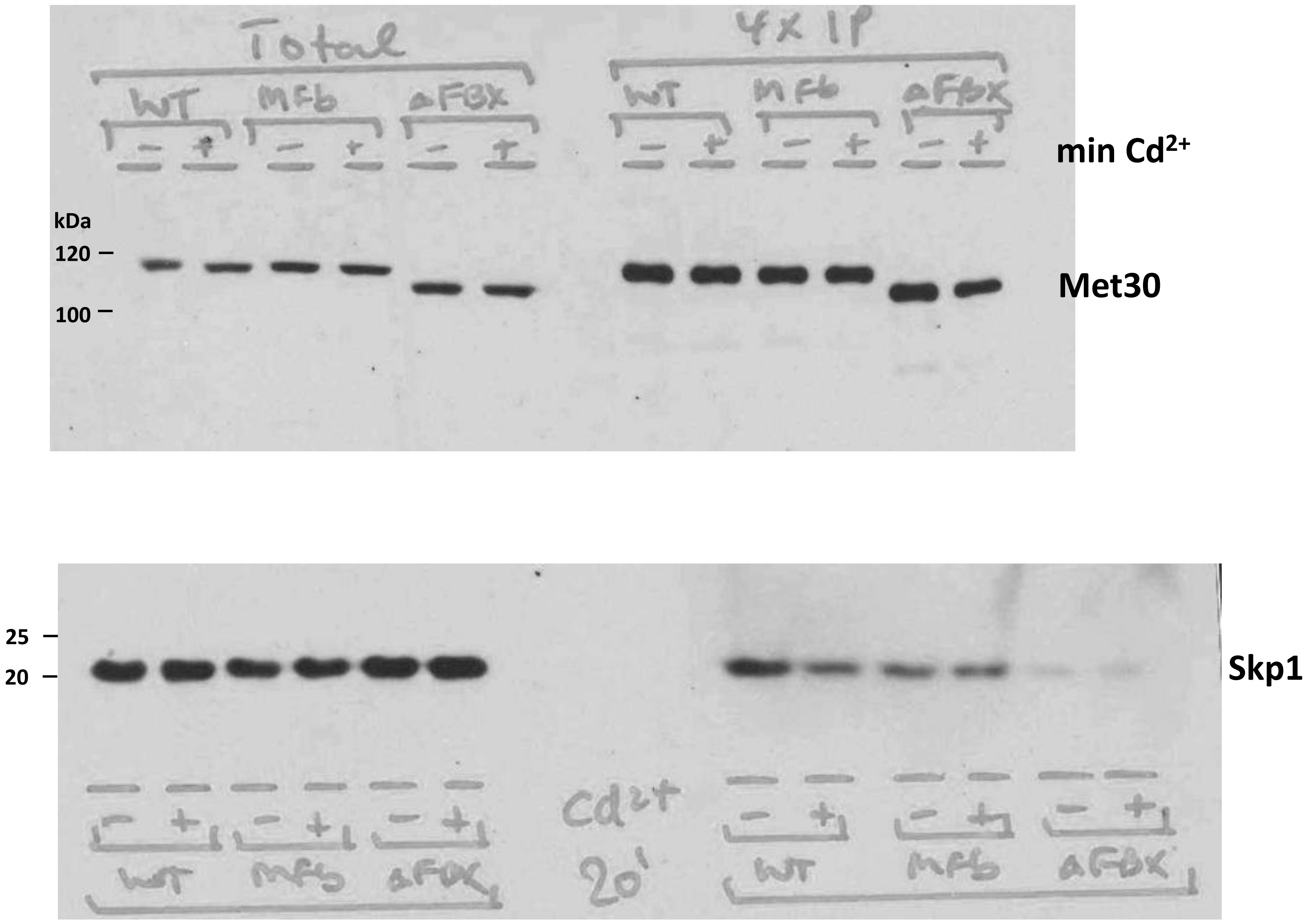

Suppl. Figure 3

A

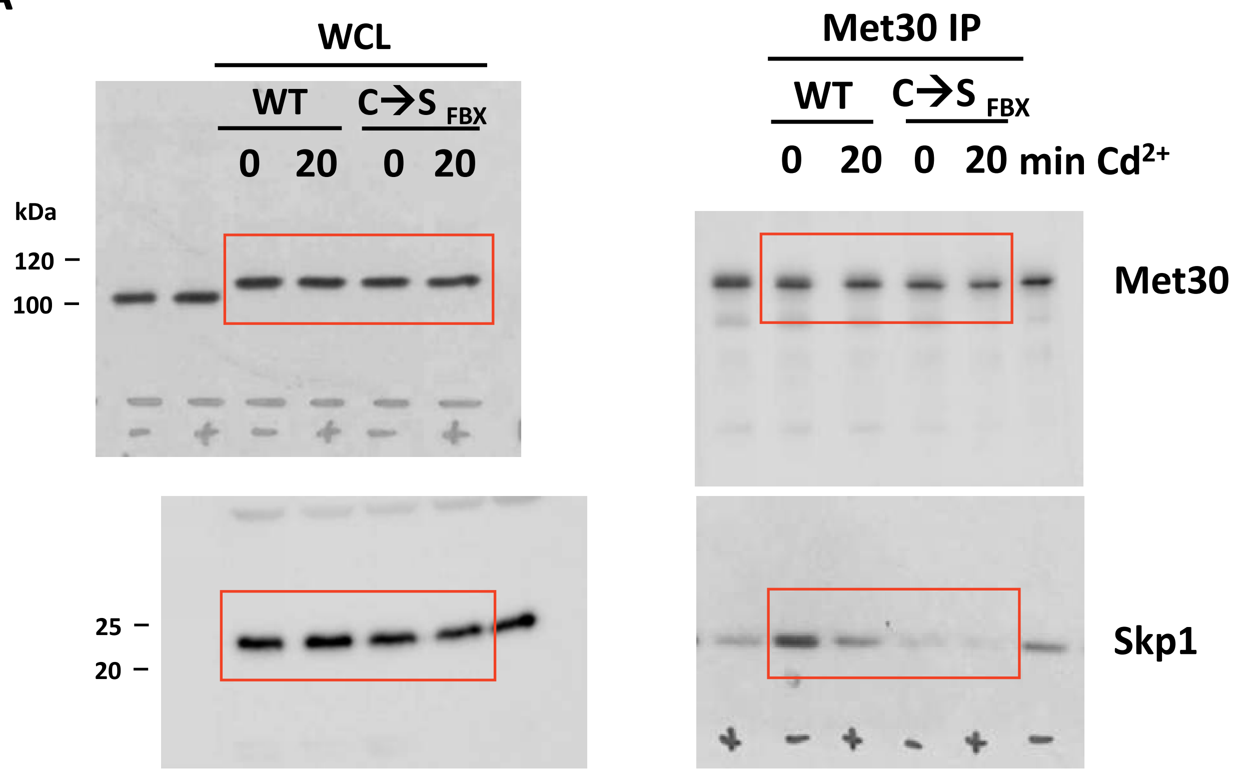

Suppl. Figure 3
D

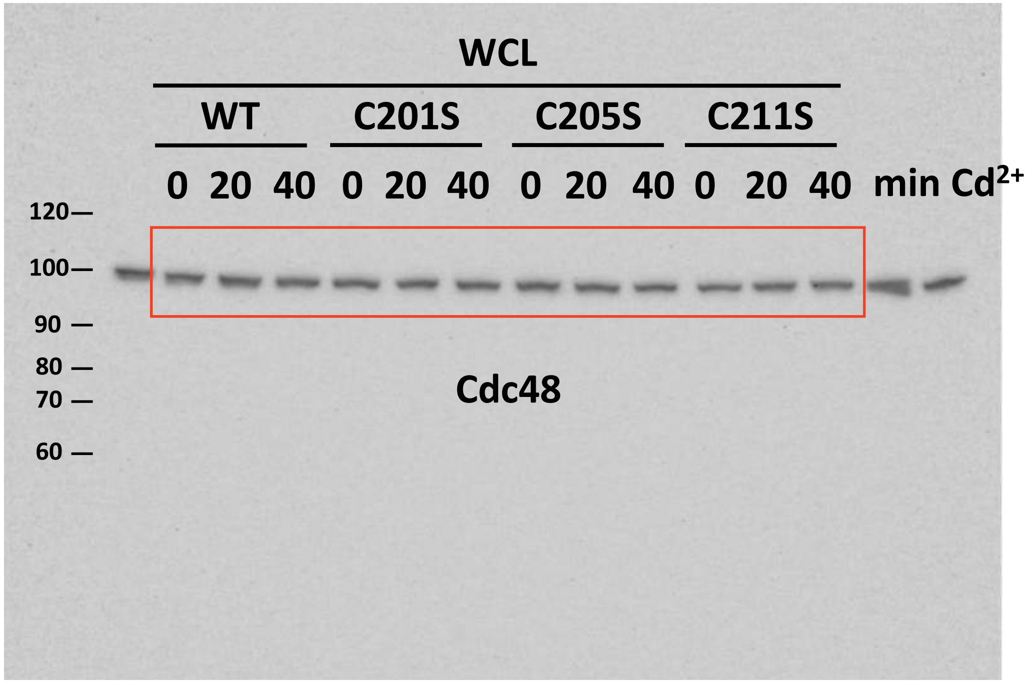

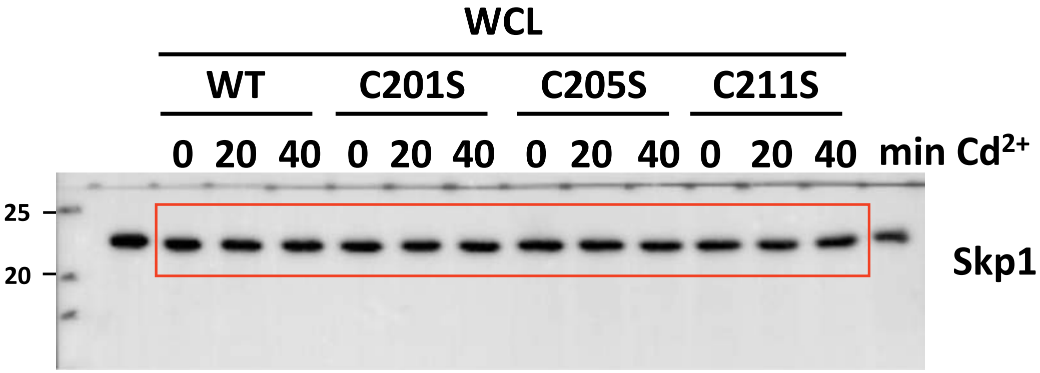

Suppl. Figure 3

F

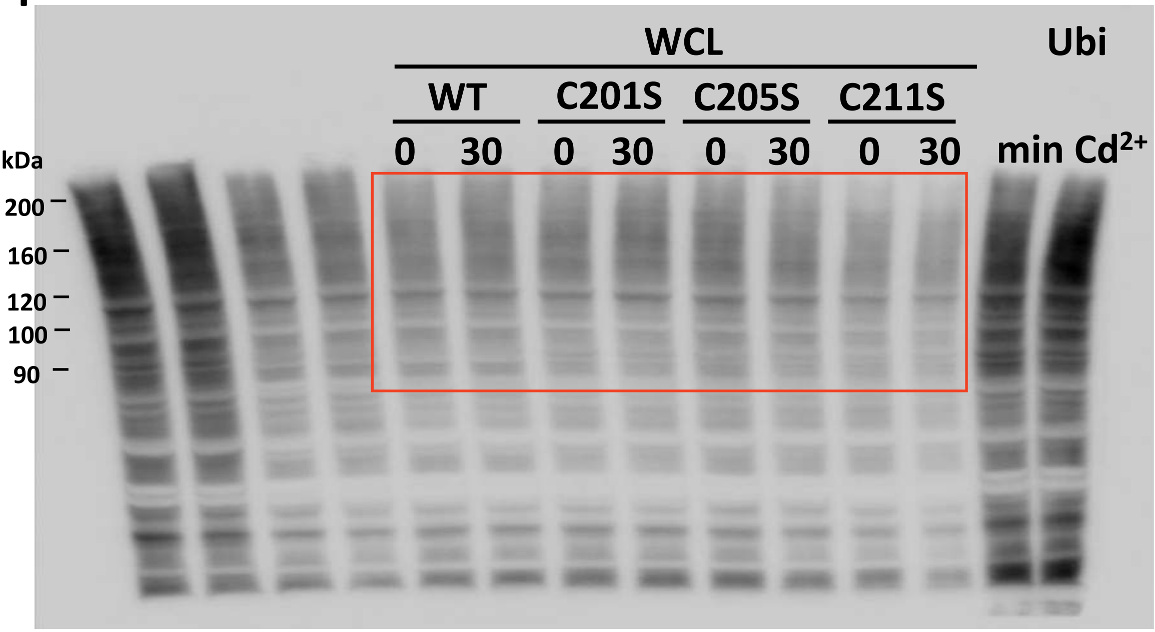

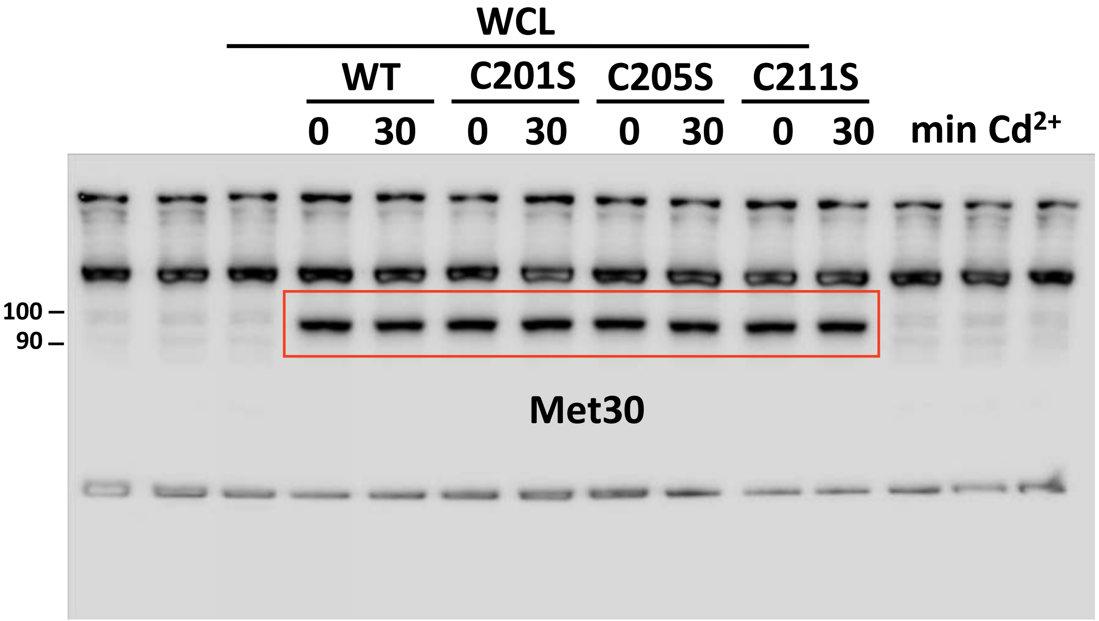

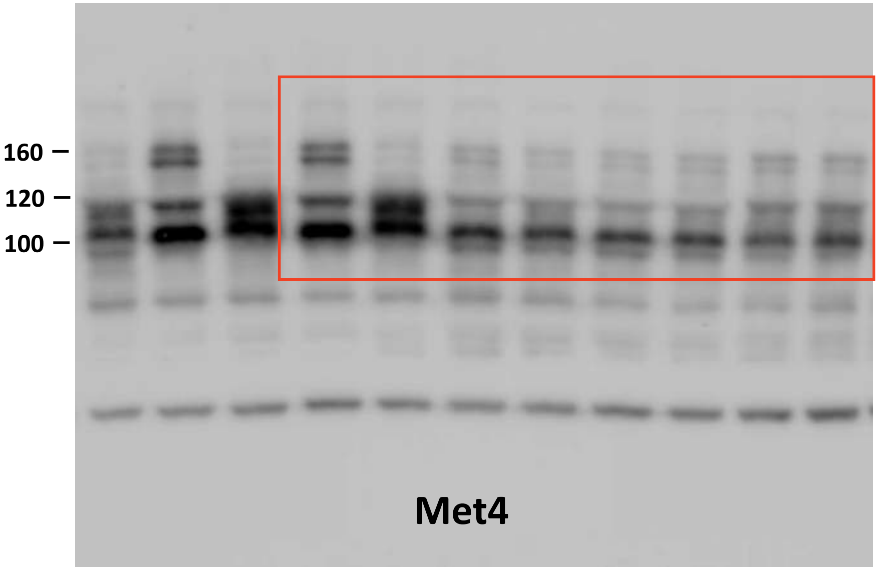

Suppl. Figure 3

G

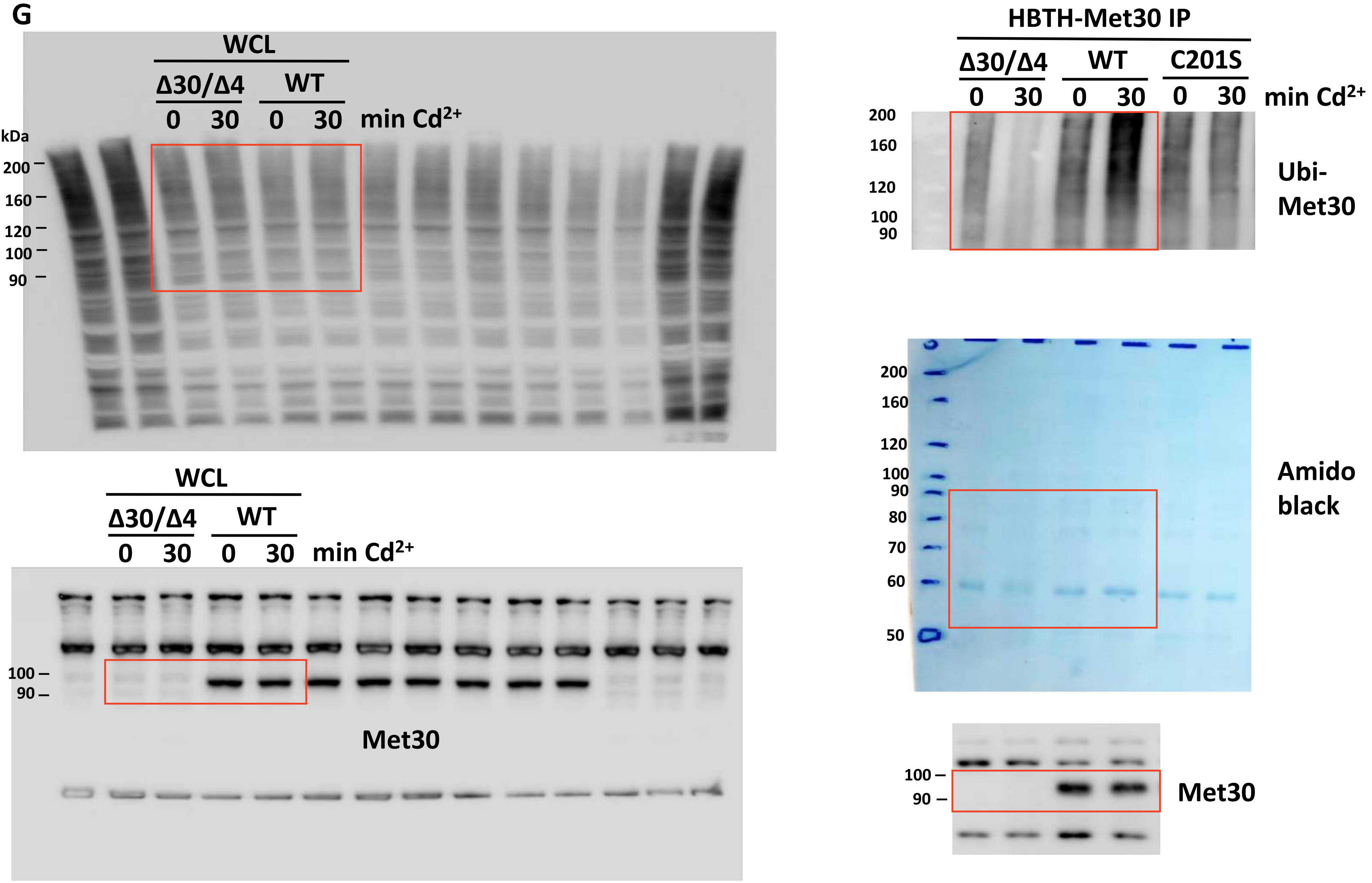

Suppl. Figure 4

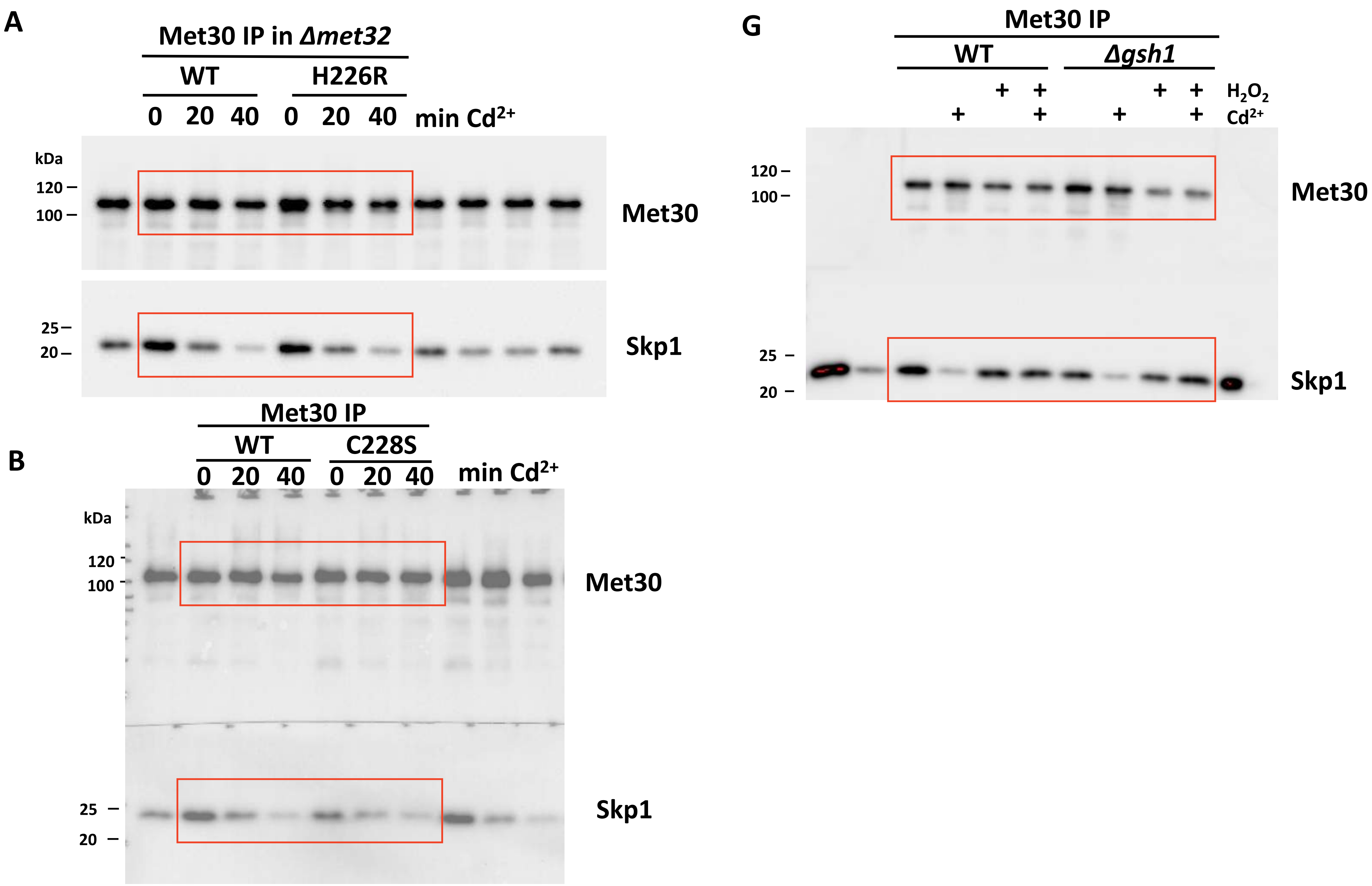

Suppl. Figure 5

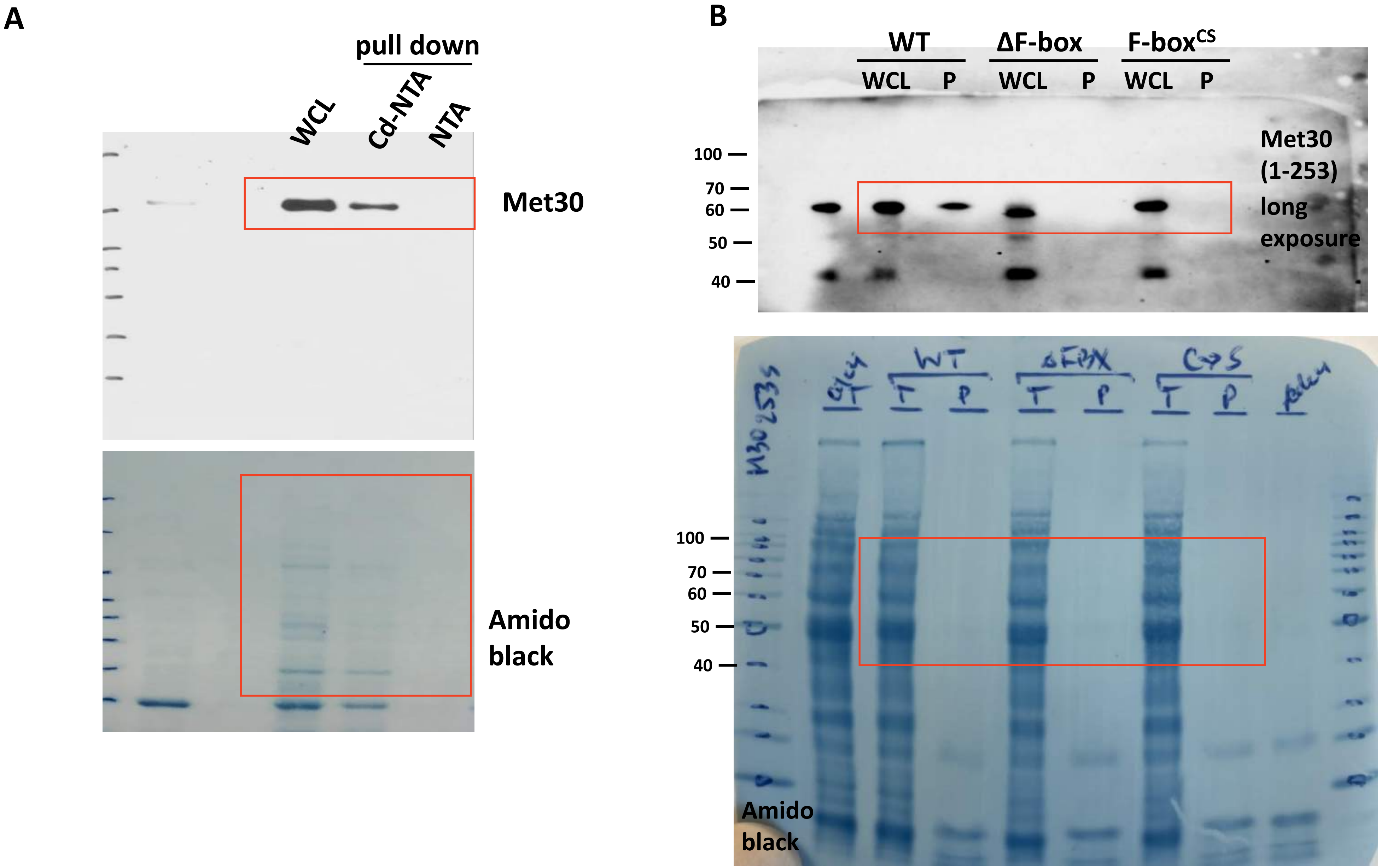

Suppl. Figure 5

C

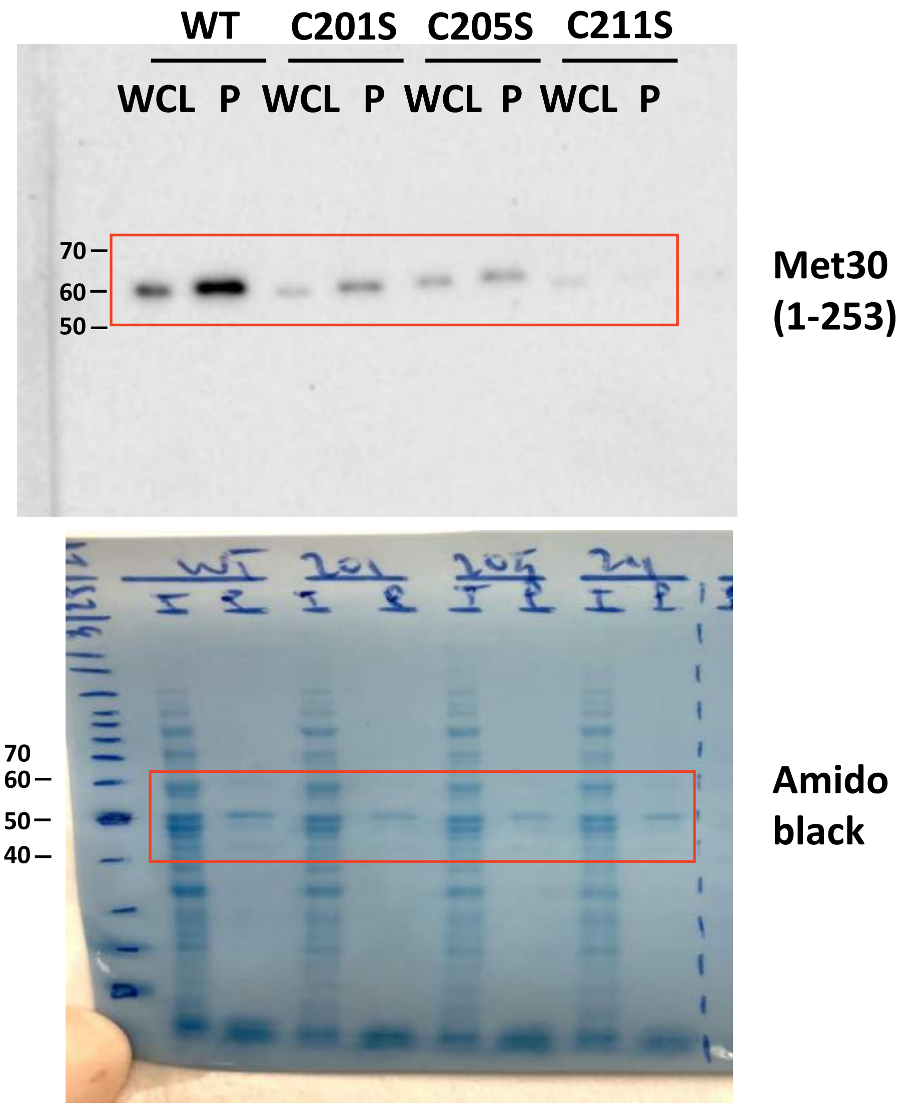

E

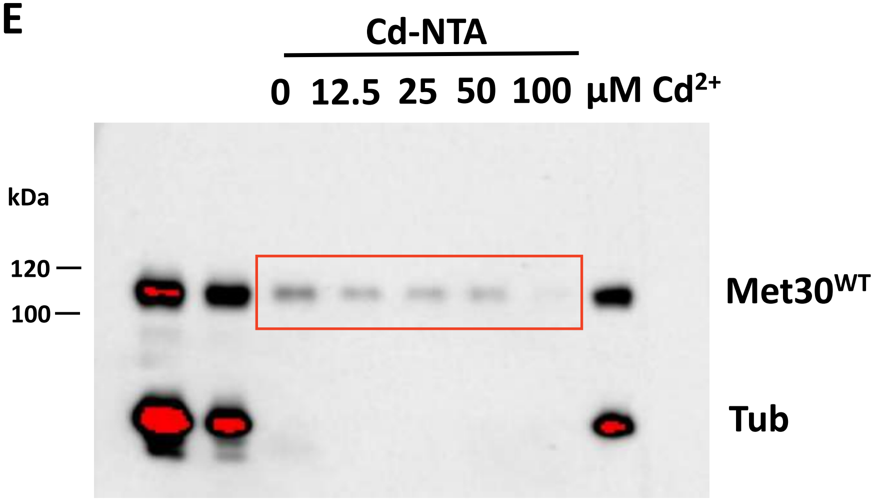

Suppl. Figure 6

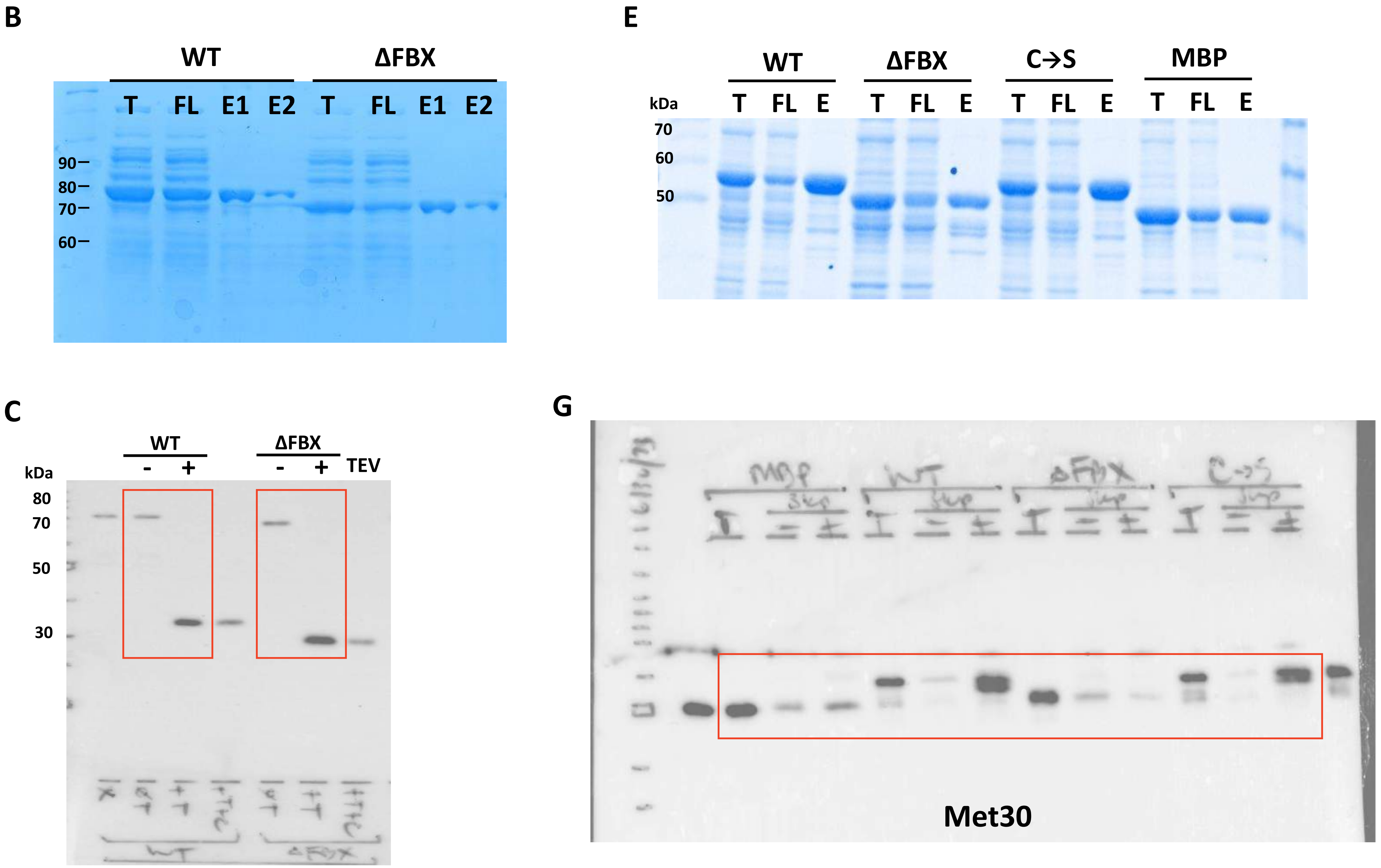

Suppl. Figure 6

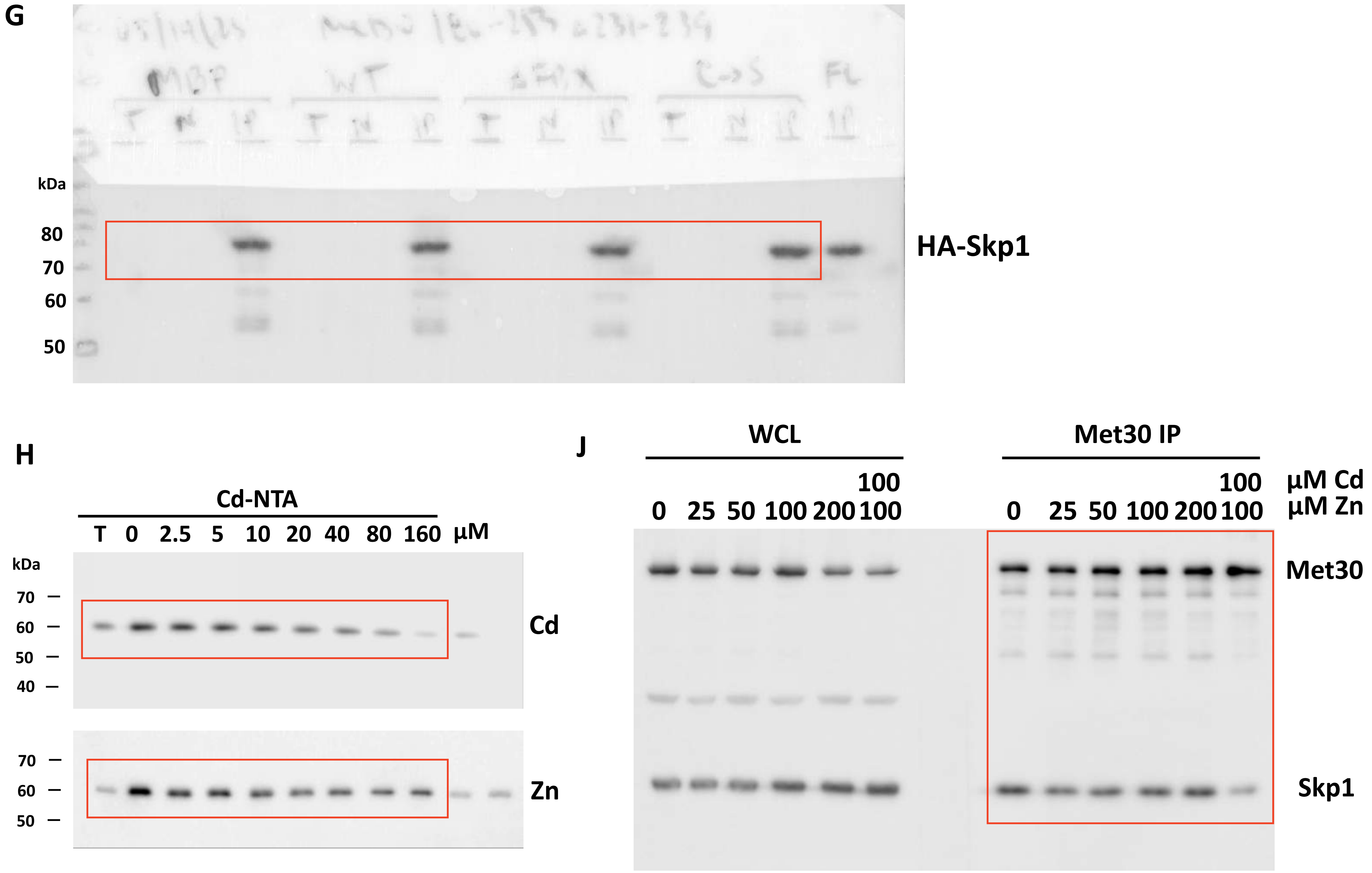

Supplement: Supplementary file 6 — Source Data [file 41467_2024_48184_MOESM6_ESM.zip › uncropped blots.pdf]
